# From beverage waste to electrical withstand: crystal-phase-controlled recycled alumina nanofillers for surface charge regulation in HVDC epoxy spacers

**DOI:** 10.1039/d6ra05417j

**Published:** 2026-09-25

**Authors:** Mahmoud Ezzat, M. Ramadan, Musa A. Said, Hany M. Abd El-Lateef, Mousa. A. Abd-Allah, S. M. A. El-Gamal, Abdelrahman Said

**Affiliations:** a Department of Electrical Engineering, Faculty of Engineering at Shoubra, Benha University Cairo 11672 Egypt abdelrahman.ghoniem@feng.bu.edu.eg; b Chemistry Department, Faculty of Science, Ain Shams University Cairo 11566 Egypt M.Ramadan@Sci.asu.edu.eg; c Department of Chemistry, Faculty of Science, Islamic University of Madinah Madinah 42351 Saudi Arabia; d Department of Chemistry, College of Science, King Faisal University Al-Ahsa 31982 Saudi Arabia hmahmed@kfu.edu.sa; e Faculty of Computer Science, Benha National University (BNU) Al Obour Egypt abdelrahman.ghoniem@cs.bnu.edu.eg

## Abstract

Epoxy spacers in gas-insulated substations (GIS) and transmission lines (GITL) accumulate surface charge under HVDC stress, distorting the electric field and lowering flashover voltage. Here, γ-rich alumina and α-Al_2_O_3_ nanofillers were synthesized from recycled aluminum beverage cans through acid dissolution, carbonate precipitation, and phase-selective calcination at 600 °C and 1200 °C, then functionalized with 3-aminopropyltriethoxysilane (APTES) and dispersed in epoxy at 1–7 wt%. Using a common recycled precursor provides a controlled basis for comparing the phase-dependent behavior of γ-rich alumina and α-Al_2_O_3_. Surface resistivity, surface potential mapping, isothermal surface potential decay (ISPD) trap analysis, DC flashover testing, and coupled electrothermal COMSOL simulation were performed. Among the investigated loadings, 5 wt% produced the highest surface resistivity for both alumina phases and was therefore selected for detailed electrical characterization. The γ-rich phase, with its defective spinel structure, gave the highest surface resistivity gain (68.1%) and mean flashover voltage (13.49 kV, +12.8% *versus* neat epoxy), whereas the corundum-structured α phase showed faster early charge dissipation (44.0% within 15 min) from a shallow-trap density more than double that of γ-rich alumina. Simulation showed γ-rich alumina cut accumulated charge by 61.8% and the peak triple-junction field by 23.1% after 120 s. The results demonstrate that calcination-induced structural modification of recycled alumina provides a controllable and sustainability-driven route for regulating HVDC surface-insulation performance.

## Abstract 2

Controlling the crystal phase of recycled alumina tunes the interfacial trap distribution, governing surface charge dissipation and flashover suppression in HVDC epoxy spacers.

## Graphical abstract

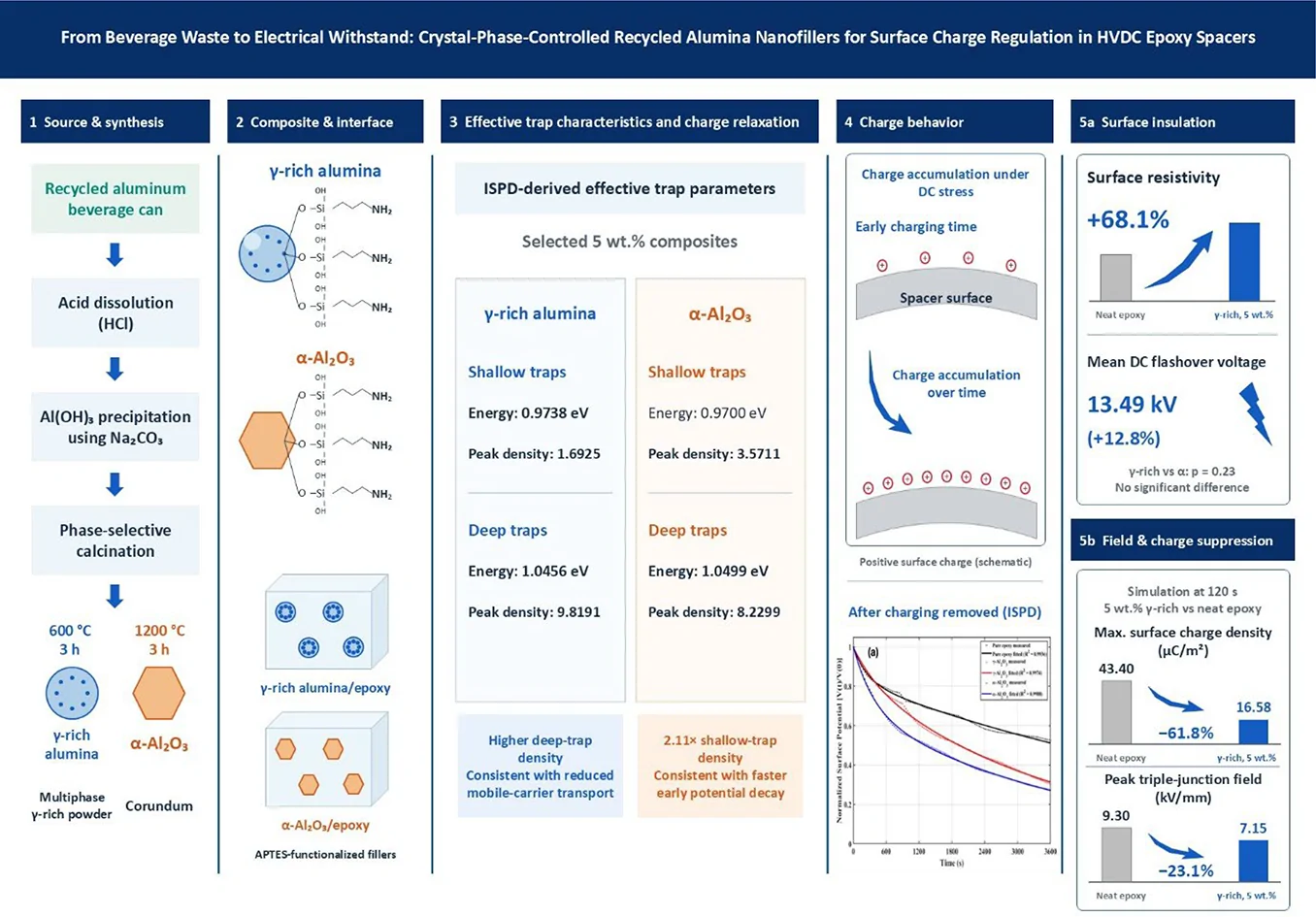

## 1. Introduction

The global expansion of high-voltage direct current (HVDC) transmission networks has brought the surface insulation performance of epoxy spacers in gas-insulated substations (GIS) and gas-insulated transmission lines (GITL) under sustained scrutiny. Because spacer reliability is ultimately governed by the chemistry of the polymer–filler interphase and the trap state it introduces, materials-design strategies have increasingly focused on tailoring filler composition and structure. Unlike alternating current systems, where periodic voltage reversal facilitates the redistribution of displaced charge carriers, direct current operation permits the uninterrupted accumulation of charge at the gas–solid interface of spacer surfaces.^1–3^ This accumulated surface charge has been identified as a primary driver of electric-field distortion in HVDC GIS insulation, and surface flashover, which is the most critical failure mode in such systems, has been directly linked to this field distortion.^1,4,5^

Research on modified epoxy dielectrics has predominantly focused on bulk electrical and thermal properties, particularly dielectric breakdown strength and thermal conductivity.^6,7^ For example, molecular ordering in epoxy/organic-acceptor composites has recently been employed to enhance both properties.^8^ Comparatively less attention has been devoted to the surface-insulation response of modified epoxy, including surface resistivity, charge trapping and dissipation, and DC flashover at gas–solid interfaces.

Surface charge accumulates at the spacer surface through several concurrent mechanisms, including bulk conduction through the solid dielectric under the applied DC field, ion drift from gas-phase ionization near electrode surfaces, and interfacial charging at the conductor–spacer contact zone.^1^ The resulting charge distribution is spatially non-uniform at the triple-junction region, where the high-voltage conductor, the insulating gas, and the solid spacer surface converge, surface charge concentrates and produces localized electric-field amplification that exceeds the macroscopic average, intensifying with both time and the thermal gradient imposed across the spacer.^9^ The polarity and magnitude of charge accumulation near the triple junction are governed by which conduction pathway, bulk solid or gas-side ionic is dominant under a given set of operating conditions.^10^

At the microstructural level, surface charge accumulation and retention are governed by the energy distribution of localized carrier trap states within the polymer matrix.^11–13^ Surface modifications that convert deep trap states to shallower ones have been shown to augment surface conductivity, accelerate charge dissipation, and raise the DC flashover threshold, with these effects quantified by two-parameter Weibull statistical analysis.^14,15^ The trap energy distribution is experimentally accessible through the isothermal surface potential decay (ISPD) method, in which the surface potential on a corona-charged specimen is monitored over time and its decay profile yields the characteristic energies and densities of the trap states.^13^ This approach has been extended to nanoscale spatial resolution by combining ISPD with Kelvin probe force microscopy, confirming that nanoparticle incorporation modifies the trap density at individual polymer–filler interfaces.^16^

Surface flashover along the gas–solid interface under DC stress has been reviewed comprehensively with respect to both governing mechanisms and material modification strategies.^4,17^ Among the material variables that determine flashover performance, surface resistivity controls charge mobility along the spacer surface while the trap distribution governs the rate of charge dissipation. These variables are coupled: modifications that alter the trap depth distribution simultaneously change the effective surface conductivity, producing coordinated effects on both charge accumulation and flashover performance.^14^

Alumina (Al_2_O_3_) nanofillers have been studied as modifiers of both the dielectric response and the space charge behavior of epoxy composites for high-voltage applications.^11,18^ The dielectric response of epoxy–alumina nanocomposites is sensitive to both the primary particle size, which determines the available interfacial area per unit volume, and the post-cure temperature, with both variables producing measurable effects on permittivity.^19^ Under combined electric and thermal fields, the Al_2_O_3_ loading level and the applied thermal gradient jointly govern the rate and spatial distribution of space charge accumulation in epoxy/Al_2_O_3_ composites.^20^ Amino-silane surface-functionalized Al_2_O_3_ nanoparticles have been reported to increase the volume resistivity and thermal conductivity by up to 17% relative to the unfilled matrix.^21^

The crystallographic phase of Al_2_O_3_ nanoparticles constitutes a material variable whose influence on the surface insulation performance of epoxy composites for HVDC GIS applications has not been investigated. The γ phase, formed below approximately 800 °C, adopts a defective spinel-like structure in which Al^3+^ cations are distributed across both tetrahedral and octahedral interstitial sites of a face-centered cubic oxygen sub-lattice, while the α phase forms above approximately 1100 °C through a reconstructive transformation producing the corundum structure, with Al^3+^ occupying exclusively octahedral positions.^22^ Machine-learning-guided density functional theory analysis of γ-rich alumina confirmed that octahedral vacancies in this phase deviate from the conventional spinel arrangement, establishing the intrinsic atomic-level disorder that distinguishes it from the fully ordered α structure.^23^ These structural differences translate into differences in specific surface area, surface hydroxyl group configuration, and the character of the polymer–filler interphase formed during composite preparation. Whether these phase-specific characteristics produce measurable differences in trap energy distributions, surface charge dissipation kinetics, and DC flashover performance has not been reported.

Both alumina phases in this study were synthesized from recycled aluminum beverage cans. γ-Al_2_O_3_ produced from waste aluminum foil through acid dissolution, sodium carbonate precipitation, and calcination has been characterized as possessing a specific surface area of 149.9 m^2^ g^−1^.^24^ Aluminum oxide phases have similarly been obtained from waste aluminum foil *via* calcination-based routes, confirming the viability of recyclable aluminum waste as a precursor for phase-selective alumina synthesis.^25,26^ Both calcination-derived alumina powders were prepared from the same recycled precursor using identical chemical processing before calcination. Thus, calcination temperature was the intentionally varied synthesis parameter. However, changing the calcination temperature simultaneously altered the phase assemblage, crystallinity, residual hydroxide content, defect structure, particle dimensions, and surface characteristics of the resulting powders. Accordingly, the present study compares two contrasting calcination-derived alumina, a partially crystalline, multiphase γ-rich material and a highly crystalline α-Al_2_O_3_ material.

This study presents a systematic multi-scale comparison of γ-rich alumina and α-Al_2_O_3_/epoxy nanocomposites at 1–7 wt% for HVDC GIS spacer surface insulation, encompassing: (i) surface resistivity screening across all loadings; (ii) surface potential mapping for charge accumulation and dissipation dynamics; (iii) ISPD-based trap energy characterization; (iv) DC surface flashover testing with Weibull statistical analysis; and (v) coupled electrothermal COMSOL simulation of a GIS spacer geometry. Results are interpreted through an integrated mechanistic interpretation connecting crystal phase characteristics to trap energy distributions and macroscopic insulation performance.

In our previous work, Zn/Al-LDH/epoxy nanocomposites were investigated as a layered nanofiller system for improving the surface charge regulation and flashover performance of GIS spacer insulation under HVDC stress.^27^ That study demonstrated that the lamellar LDH structure can enhance surface resistivity, promote surface charge relaxation through trap modulation, and reduce electric-field distortion at the spacer level. However, the enhancement mechanism in that system was primarily governed by the unique layered morphology and tunable interfacial chemistry of Zn/Al-LDH.

This study makes three original contributions. First, it establishes an association between the calcination-derived structural state of recycled alumina and the surface-insulation performance of epoxy nanocomposites while maintaining a common precursor and identical functionalization and composite-processing procedures. The comparison encompasses the combined effects of phase assemblage, crystallinity, defect structure, particle morphology, and surface/interphase characteristics rather than an isolated pure-phase effect. Second, it identifies and distinguishes two complementary enhancement mechanisms: surface resistivity as the primary flashover-governing mechanism and trap-mediated charge dissipation as a supporting mechanism whose relative importance increases under sustained DC service conditions through a systematic integrated mechanistic interpretation spanning surface resistivity, ISPD trap characterization, surface potential mapping, and Weibull flashover statistics. Third, it provides a proof-of-concept system-level assessment using an idealized 2D axisymmetric GIS spacer geometry. Collectively, the findings demonstrate that phase-selective calcination of a single recycled waste stream constitutes a controllable and sustainable design strategy for performance-optimized HVDC insulation materials.

## 2. Materials and methods

### 2.1 Materials

The epoxy matrix was a commercially supplied two-component electrical-insulation system, KEMAPOXY 150 JM (Chemicals for Modern Building International, Giza, Egypt). The system comprises KEMAPOXY 150 JM (A), a medium-viscosity DGEBA-type liquid epoxy resin produced from bisphenol A and epichlorohydrin, with a reported epoxy equivalent weight of approximately 185 g eq^−1^, and KEMAPOXY 150 JM (B), a difunctional primary-amine curing agent based on polyoxypropylenediamine, with an average molecular weight of approximately 230 g mol^−1^.^28^ Components A and B were combined at the manufacturer-recommended mass ratio of 3 : 1. According to the manufacturer's technical datasheet, the mixed system has a density of 1.15 ± 0.02 kg L^−1^ at 25 °C and a pot life of approximately 45 min. Its initial and final setting times are 10–12 h and 24 h, respectively, whereas full hardness under ambient curing conditions is reached after 7 days. Supporting chemicals used in nanofiller synthesis and functionalization included concentrated hydrochloric acid (HCl, 37%, Sigma-Aldrich, St. Louis, USA), sodium carbonate (Na_2_CO_3_, ≥99.5%, Sigma-Aldrich, St. Louis, USA), 3-aminopropyltriethoxysilane (APTES, 99%, Sigma-Aldrich, St. Louis, USA), and absolute ethanol (99%, El Nasr Pharmaceutical Chemicals, Cairo, Egypt), all used as received without further purification.

### 2.2 Synthesis of recycled alumina nanofillers

Aluminum beverage cans were subjected to steam treatment at approximately 100 °C for 30–45 minutes to loosen the exterior printed coating, followed by surface wiping with acetone to remove residual organic material. The end sections of the cans, which contain alloy compositions different from the cylindrical body, were removed by cutting. The remaining aluminum body was mechanically chopped into small fragments to maximize the effective contact surface area for subsequent dissolution.

Five grams of pre-treated aluminum fragments were placed into 100 mL of distilled water and 60 mL of concentrated HCl (37%) was added dropwise under continuous mechanical stirring at 300 rpm over approximately 15 minutes, allowing controlled evolution of hydrogen gas. The resulting aluminum chloride solution was filtered to remove any undissolved material. A 2 M Na_2_CO_3_ solution was then added dropwise under continuous stirring, with the pH monitored and maintained in the range 7–8 to achieve selective precipitation of Al (OH)_3_ while minimizing re-dissolution. The reactions are shown in eqn (1) and (2):

1 2Al(s) + 6HCl(aq) → 2AlCl_3_(aq) + 3H_2_(g)

2 2AlCl_3_(aq) + 3Na_2_CO_3_(aq) + 3H_2_O → 2Al(OH)_3_(s) + 6NaCl(aq) + 3CO_2_(g)

Stirring was continued for 1 hour after complete addition to ensure thorough precipitation. The Al (OH)_3_ precipitate was recovered by filtration and washed with successive portions of distilled water until the filtrate reached neutral pH, confirming the removal of residual chloride and sodium ions. The washed precipitate was dried at 80 °C for 12 hours and ground to a fine powder.

The dried Al (OH)_3_ powder was calcined in a programmable muffle furnace in static air using a controlled heating rate of 5 °C min^−1^. A 3 hour isothermal dwell was applied at each target temperature. Calcination at 600 °C yields γ-rich alumina through dehydration of the hydroxide precursor, while calcination at 1200 °C drives the complete reconstructive phase transformation to α-Al_2_O_3_ in the corundum structure as shown in eqn (3) and (4):^22,24^3
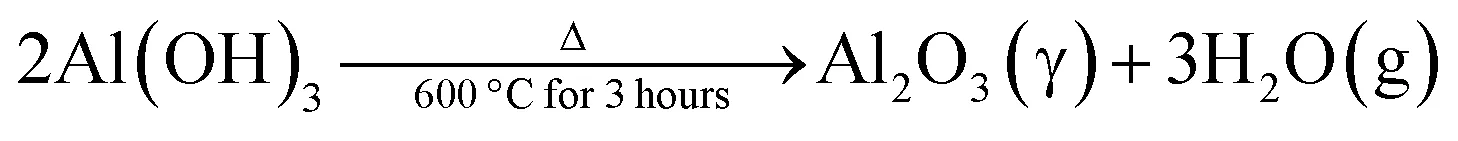
4
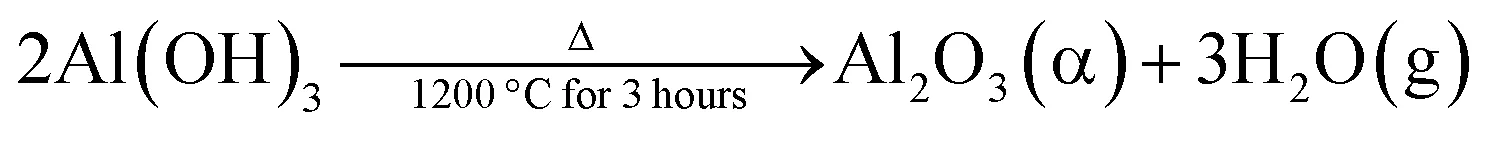


Experimental product yields were 8.4 g for γ-rich alumina and 8.1 g for α-Al_2_O_3_, corresponding to yields of 89.4% and 86.2% based on the theoretical stoichiometry. The lower yield for α-Al_2_O_3_ is consistent with greater material loss during the prolonged high-temperature dwell.

**Fig. 1:**
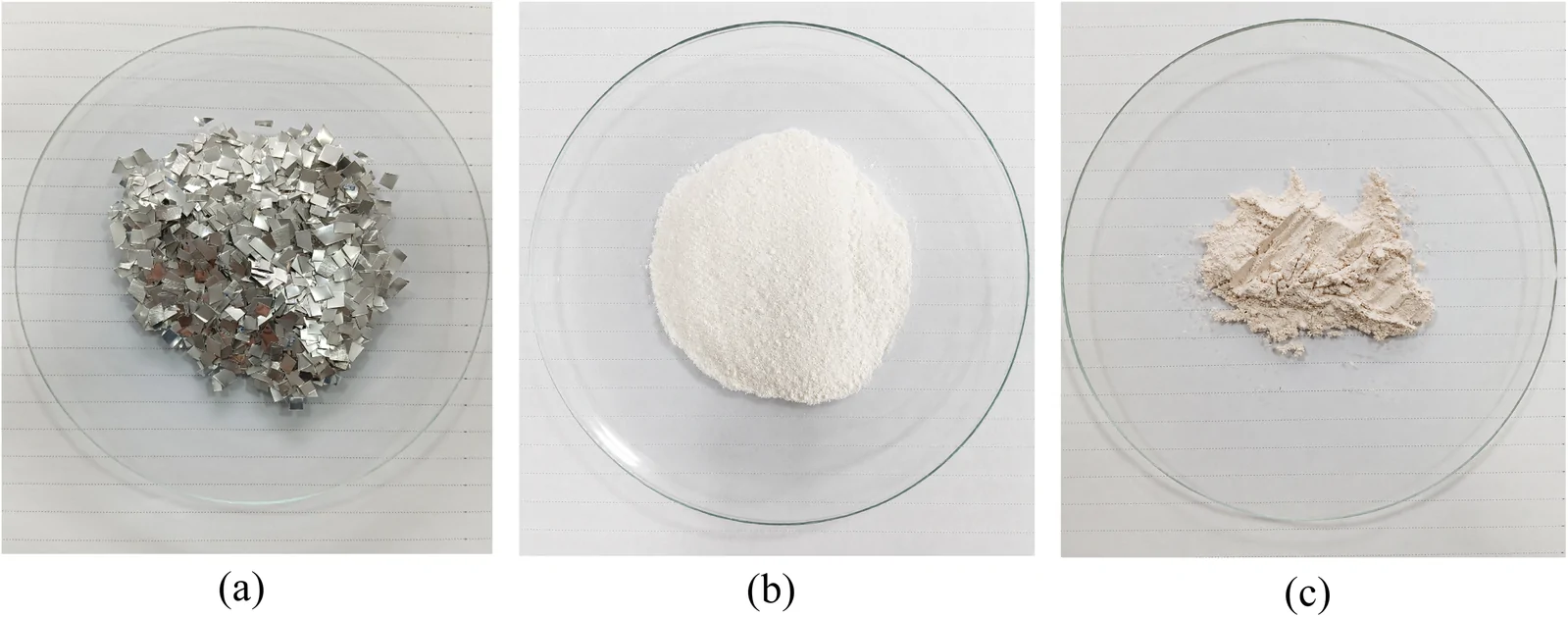
Photographic images illustrating the main stages of recycled alumina preparation: (a) shredded aluminum beverage-can, (b) dried aluminum hydroxide/alumina, and (c) calcined alumina powder.

The visual transformation of the recycled aluminum precursor into alumina powder is presented in Fig. 1, which summarizes the main physical stages of the synthesis process from shredded beverage-can fragments to the final calcined alumina product.

### 2.3 APTES surface functionalization

**Fig. 2:**
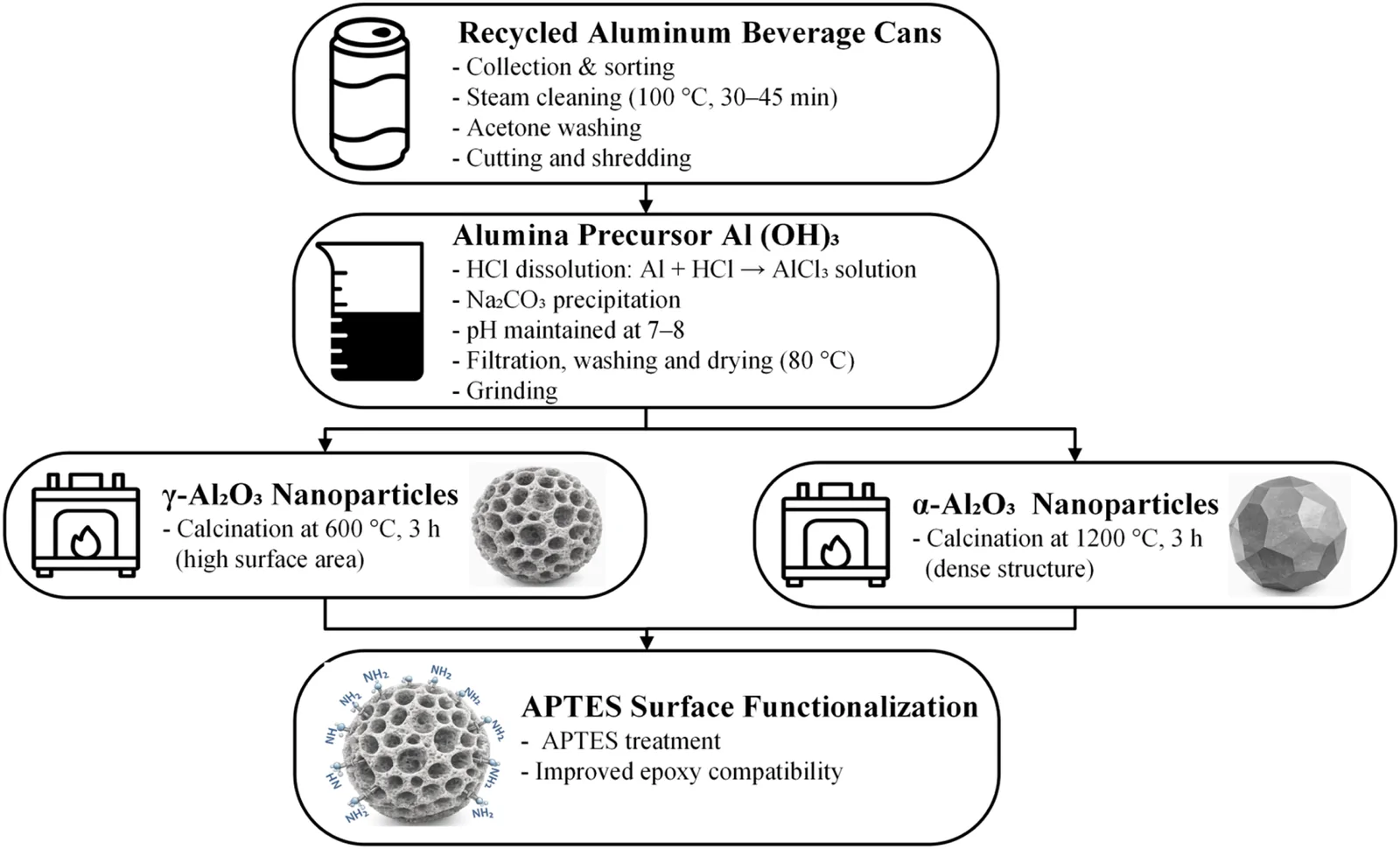
Schematic illustration of the synthesis and APTES functionalization of recycled γ-rich alumina and α-Al_2_O_3_ nanoparticles derived from aluminum beverage cans.

Both alumina phases were surface-functionalized with 3-aminopropyltriethoxysilane (APTES) to improve compatibility with the epoxy matrix. Three grams of alumina powder were dispersed in 60 mL of absolute ethanol under ultrasonication at 70 °C for 30 minutes, followed by mechanical stirring at 500 rpm for a further 30 minutes. A 10 wt% ethanolic APTES solution containing 3.0 mL of APTES, corresponding to a fixed APTES/alumina ratio of 1.0 mL g^−1^, was introduced dropwise under continuous stirring. The same APTES/alumina ratio was used for both α- and γ-Al_2_O_3_. The mixture was maintained at 70 °C for 5 hours to allow the hydrolysis of APTES ethoxy groups and the condensation of the resulting silanol groups with surface hydroxyl sites on the alumina. The functionalized particles were recovered by filtration, washed with ethanol to remove unreacted silane, and dried at 80 °C overnight.^6,29^ The process of preparation of both phases of alumina from beverage cans and the functionalization of its surface is schematically illustrated in Fig. 2.

### 2.4 Experimental methodology

#### 2.4.1 Characterization of recycled alumina nanofillers

To confirm the phase identity and structural quality of the synthesized γ-rich alumina and α-Al_2_O_3_ powders prior to epoxy incorporation, the nanofillers were subjected to a combined diffraction and electron-microscopy characterization protocol, as detailed in our companion study.^7^ Phase composition and crystallinity were determined by X-ray diffraction (XRD, X'Pert PRO, PANalytical, Netherlands) using Cu Kα radiation (*λ* = 1.5406 Å), with patterns collected over a 2*θ* range of 5–70° at a step size of 0.02°. This analysis enabled identification of the characteristic diffraction reflections corresponding to each calcination temperature and quantification of the relative crystalline content of the γ- and α-phases.

To complement the bulk-phase information obtained from XRD, the nanoscale morphology, particle dimensions, and local crystallographic ordering of the two alumina phases were examined by high-resolution transmission electron microscopy coupled with selected-area electron diffraction (HR-TEM/SAED, 2100 Plus, JEOL, Japan), operated at an accelerating voltage of 200 kV. The SAED patterns provided direct evidence of the diffraction-spot sharpness characteristic of each phase, while particle size distributions were obtained by manually measuring a statistically representative set of nanoparticles from the HR-TEM micrographs.

To provide spectroscopic evidence of APTES functionalization, Fourier-transform infrared spectroscopy (FTIR, Unicam Mattson 1000) was performed on the γ-rich recycled alumina and α-Al_2_O_3_ powders before and after surface treatment. The spectra were recorded over the wavenumber range of 400–4000 cm^−1^ under identical measurement conditions. The comparison focused on changes in the surface hydroxyl and Al–O vibration regions and on the appearance of APTES-related bands associated with C–H, N–H, Si–O–Si, and Al–O–Si groups.

Finally, to quantitatively compare the accessible surface characteristics of the two alumina nanofillers, nitrogen adsorption–desorption isotherms were obtained using a BELSORP MINI X analyzer (Microtrac, Japan). The specific surface area was calculated using the Brunauer–Emmett–Teller (BET) method, while the pore-size distribution and total pore volume were determined using the Barrett–Joyner–Halenda (BJH) model.

#### 2.4.2 Nanocomposite fabrication

**Fig. 3:**
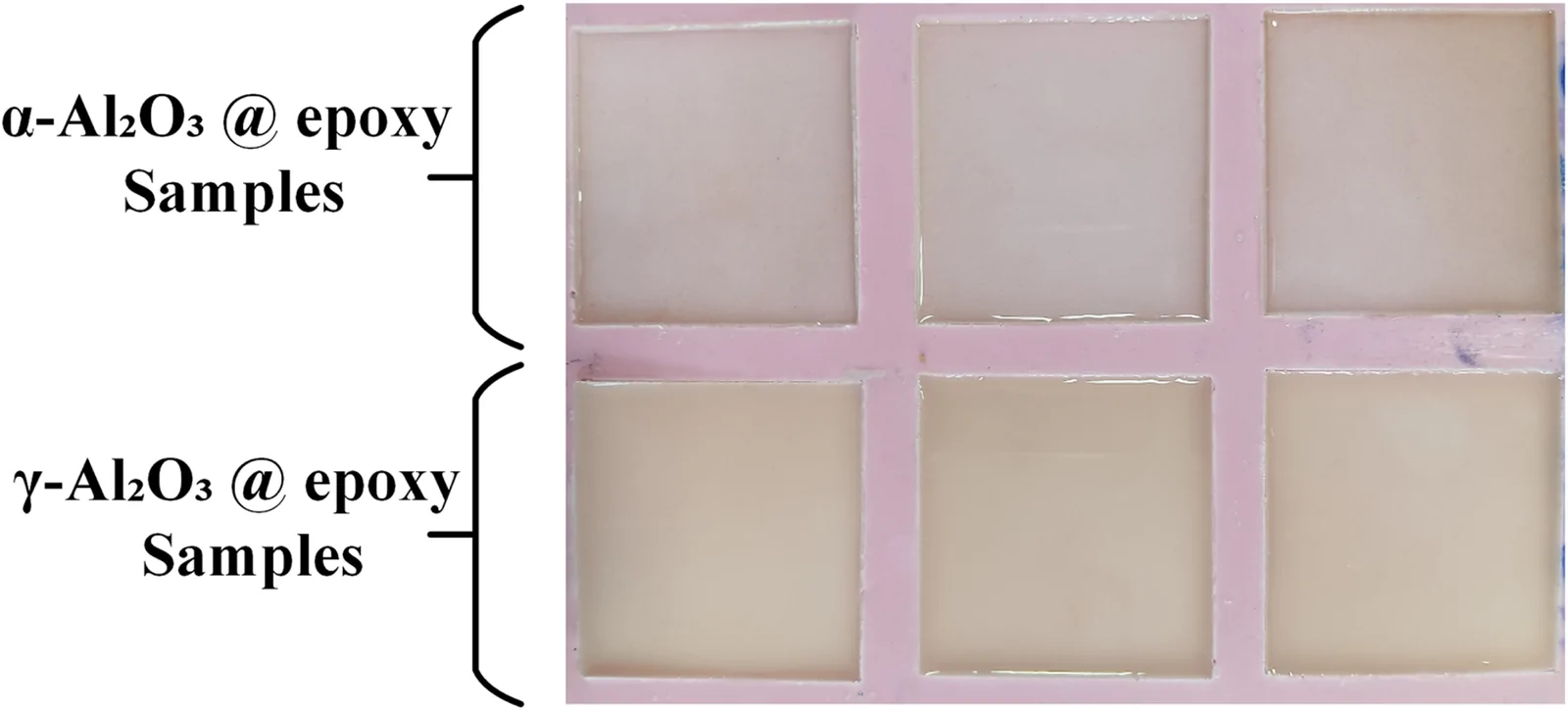
Photographs of the fabricated epoxy nanocomposite specimens containing recycled alumina nanofillers.

Epoxy nanocomposites were prepared at filler loadings of 1, 3, 5, and 7 wt% for each alumina phase, with unfilled epoxy as the reference. Functionalized alumina was dried at 80 °C for 12 hours before use. The particles were dispersed in a measured volume of absolute ethanol by ultrasonication for 60 minutes, then gradually added to the liquid epoxy resin under continuous mechanical stirring for 2 hours, followed by an additional 30 minutes of ultrasonication to ensure uniform dispersion. The ethanol was evaporated at 80 °C under stirring until constant weight was achieved. The hardener was added at a 3 : 1 (resin : hardener) weight ratio, mixed gently for 3 minutes as recommended by manufacturer's datasheet, and the mixture was degassed on a preheated plate at approximately 50 °C. Specimens were cast into silicone molds 5 cm × 5 cm with 1 mm thickness and cured at ambient temperature for 24 hours, then post-cured at 120 °C for 3 hours to ensure full crosslinking.^6,7^ While our previous study reports the bulk dielectric and thermal performance of these recycled alumina nanocomposites,^7^ the present work focuses exclusively on surface charge regulation and DC flashover performance for GIS spacer applications. All specimens were examined in their as-cast condition without grinding or mechanical polishing as shown in Fig. 3. Before the surface-resistivity and flashover measurements, the test surfaces were cleaned with ethanol and allowed to dry completely. The same surface-cleaning procedure was applied to all specimens. No separate moisture-conditioning treatment was performed; however, all specimens were stored and tested under the same laboratory conditions to minimize differences arising from moisture exposure and surface handling.

#### 2.4.3 Surface resistivity

**Fig. 4:**
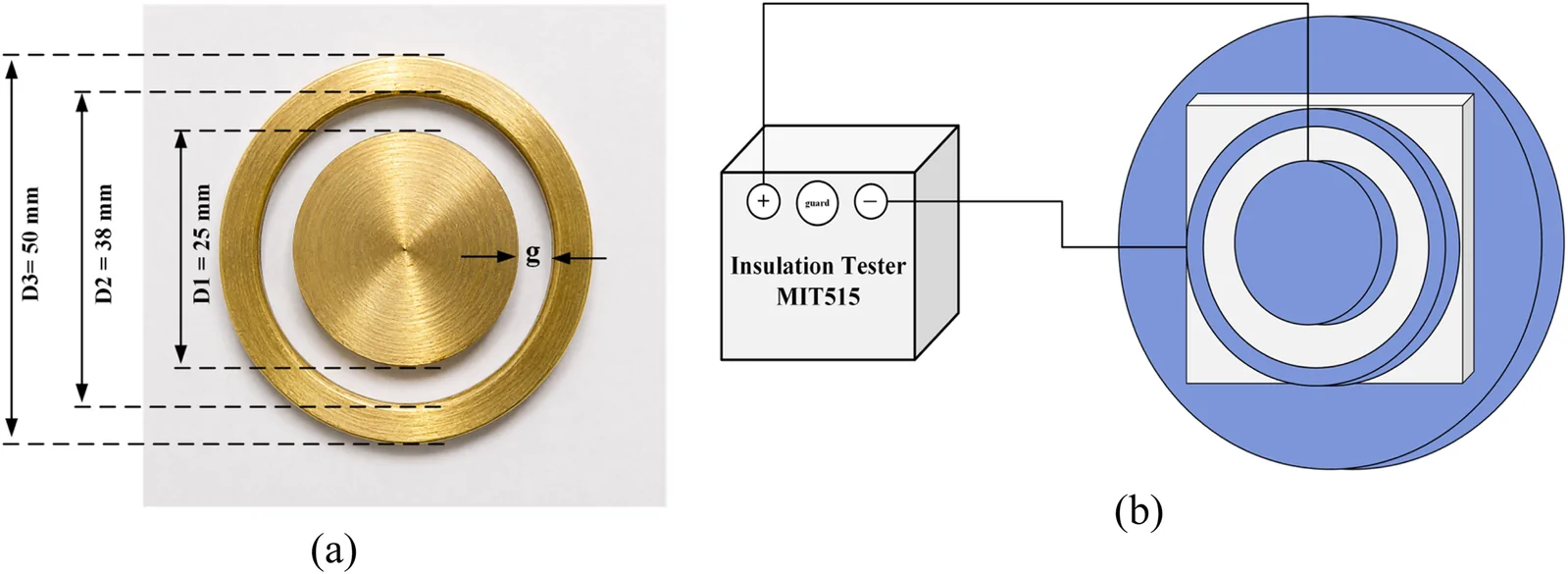
Guarded electrode configuration for surface resistivity measurement: (a) electrode dimensions and (b) experimental test setup adapted from ref. 27.

Surface resistivity was measured in accordance with ASTM D257 using a guarded electrode configuration and a high-resistance tester.^30^ This test was used as a screening stage to determine the best performance loading weight percentage among the investigated formulations to be used in the next tests. A DC test voltage of 500 V was applied between the central measuring electrode and the guard ring for 60 seconds, with the lower electrode floating to confine conduction to the specimen surface as shown in Fig. 4b. Measurements were performed at approximately 35 °C and 50% relative humidity, and resistance values were corrected to the standard reference temperature of 23 °C using an Arrhenius-type temperature correction presented in eqn (5):5
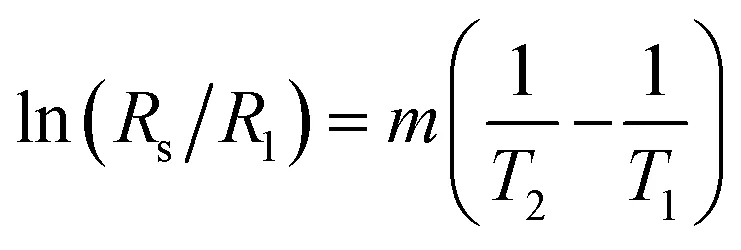
where *R*_s_ is the corrected resistance, *R*_1_ is the measured resistance, *m* is the activation constant, a common literature-based value of 9550 was applied to neat epoxy and all nanocomposites solely for temperature normalization,^31^*T*_2_ is the target temperature in kelvin, and *T*_1_ is the temperature during measurement in kelvin.

The surface resistivity *ρ*_s_ was then calculated from the corrected surface resistance *R*_s_ using eqn (6):6
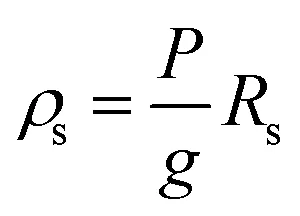
where *P* is the effective perimeter of the guarded electrode and equals π*D*_0_ and *g* is the inter-electrode gap shown in Fig. 4a. Ten independent specimens were tested per composition to provide statistical confidence in the screening results.

#### 2.4.4 Surface potential distribution and decay

**Fig. 5:**
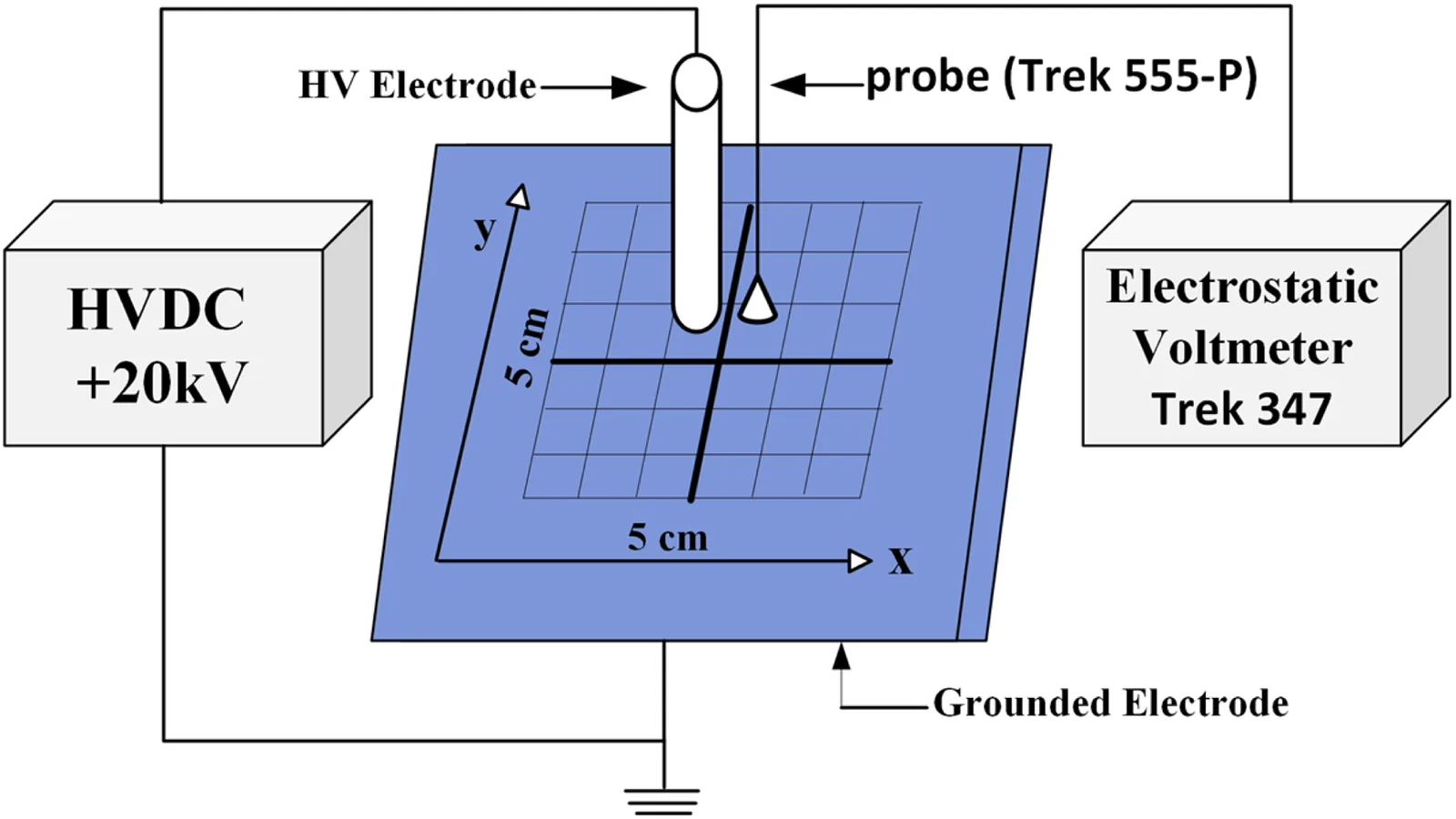
Schematic of the surface charge accumulation and dissipation measurement setup adapted from ref. 27.

Surface potential experiments were conducted on the best formulation of both phases using a rod-to-plane electrode geometry. A DC voltage of +20 kV was applied to the rod electrode 7 mm above the specimen surface for 30 minutes to accumulate charge on the specimen.^15^ Immediately after voltage removal, the surface potential distribution was mapped using a non-contact electrostatic voltmeter (Trek 347) with a high-sensitivity probe (Trek 555-P) positioned 2 mm above the specimen surface. Before each mapping measurement, the probe was positioned 2 mm above a grounded conducting reference surface, and the zero offset was adjusted. Measurements were taken at *t* = 0, 15, 30, and 60 minutes after voltage removal, The specimen surface was mapped using a uniform grid, corresponding to a center-to-center sampling interval of approximately 8.33 mm in both directions as shown in Fig. 5. According to the manufacturer's specifications, the voltage-display accuracy is better than ±0.1% of full scale. All experiments were carried out at approximately 30 °C and 56% relative humidity.

#### 2.4.5 Trap energy characterization (ISPD)

Trap energy levels and their density distributions were extracted from the 3600 s surface potential decay curves constructed using the data recorded in the previous experiment, and following the procedure adopted in ref. 13. The experimental decay curves were fitted using a dual-exponential model as presented in eqn (7):

7 *V*_s_(*t*) = *a*_1_e^−*b*_1_*t*^ + *a*_2_e^−*b*_2_*t*^

where *a*_1_ and *a*_2_ are the initial contributions of the two trap populations and *b*_1_, *b*_2_ are the decay-rate constants (s^−1^) associated with shallow and deep trap states, respectively. The trap energy *E*_*t*_ corresponding to a given time *t* in the decay process is determined by thermally activated de-trapping theory that formulated as shown in eqn (8):

8 *E*_*t*_ = *k*_B_*T* ln(*f*_ATE_*t*)

where *k*_B_ is Boltzmann's constant, *T* is the absolute temperature during measurement, and *f*_ATE_ represents the attempt-to-escape frequency, taken as 4.17 × 10^13^ s^−1^.^15^ The corresponding trap state density *Q*_*t*_ at energy *E*_*t*_ is calculated from the rate of surface potential decay as in eqn (9):9
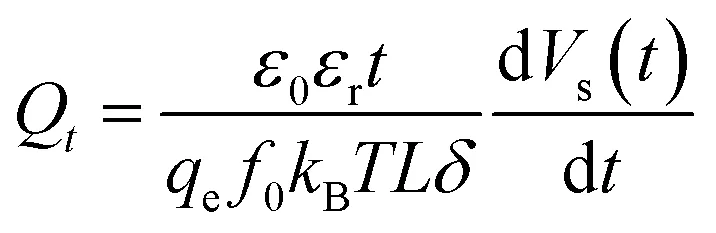
where *ε*_0_ is the vacuum permittivity, *ε*_r_ is the specimen relative permittivity, *q*_e_ is the elementary charge, *f*_0_ is the initial trap occupancy factor, *L* is the specimen thickness, and *δ* is the effective charge distribution depth. All variables taken as reported in literature.^13,32^

#### 2.4.6 DC surface flashover testing

**Fig. 6:**
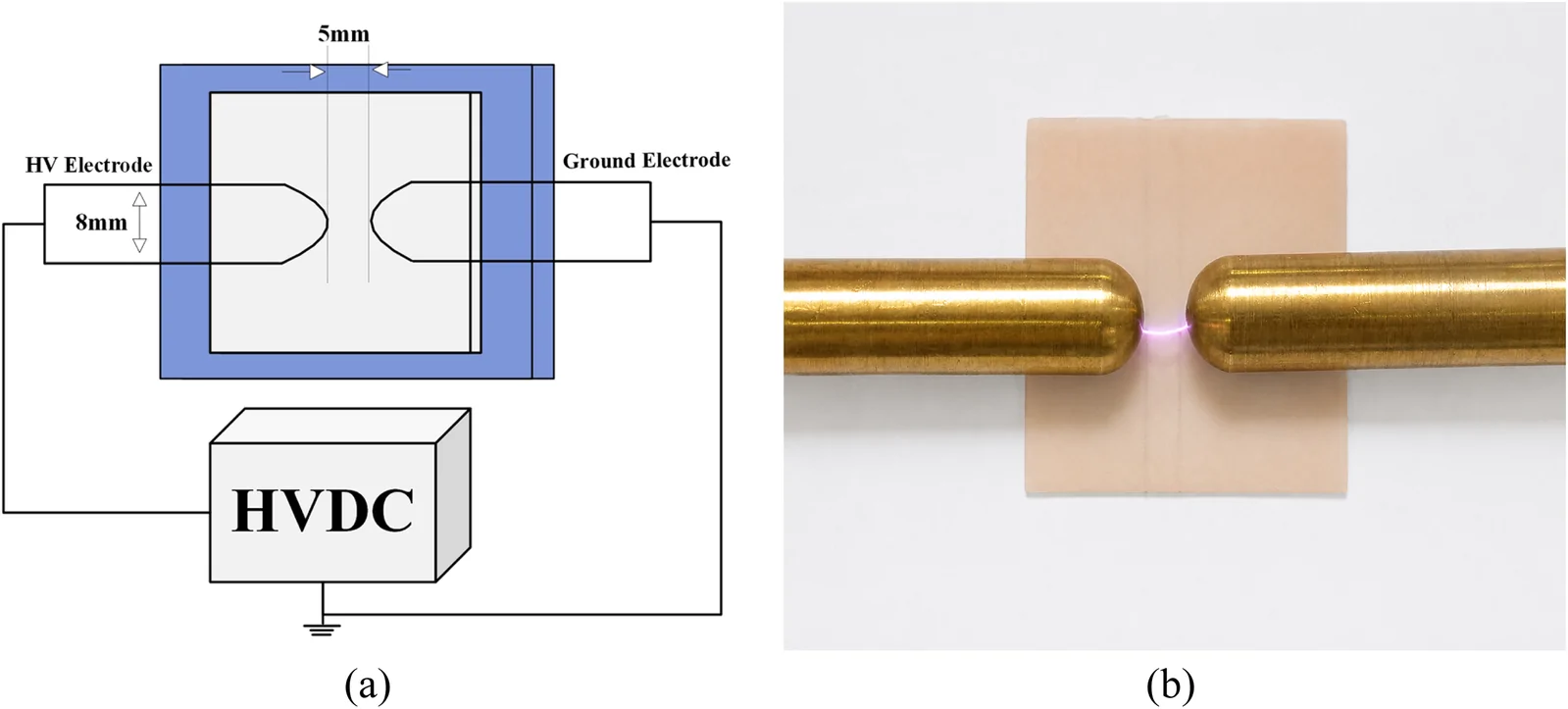
(a) DC surface flashover electrode configuration, (b) experimental photograph of the flashover discharge adapted from ref. 27.

DC surface flashover tests were conducted using a finger-type electrode configuration consisting of two semi-spherical copper electrodes (radius 8 mm, inter-electrode gap 5 mm) placed on the specimen surface as shown in Fig. 6. A DC high voltage was applied at a controlled ramp rate of 0.5 kV s^−1^ until flashover occurred, identified by a visible discharge and an abrupt voltage collapse. Each material was tested on ten independent specimens, with a 2 minute rest interval between tests to allow surface charge dissipation. The resulting flashover voltage data were analyzed using the two-parameter Weibull distribution.^14^ The cumulative failure probability is calculated using eqn (10):10
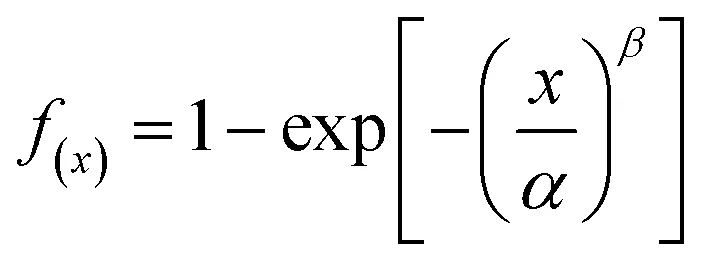
where *α* is the characteristic flashover voltage at the 63.2% failure probability and *β* is the shape parameter reflecting the statistical scatter and reliability of the results. Bootstrap 95% confidence intervals were computed for *α*, *β*, and the mean flashover voltage to visualize and quantify the reliability of the results.

#### 2.4.7 GIS spacer electrothermal simulation

**Fig. 7:**
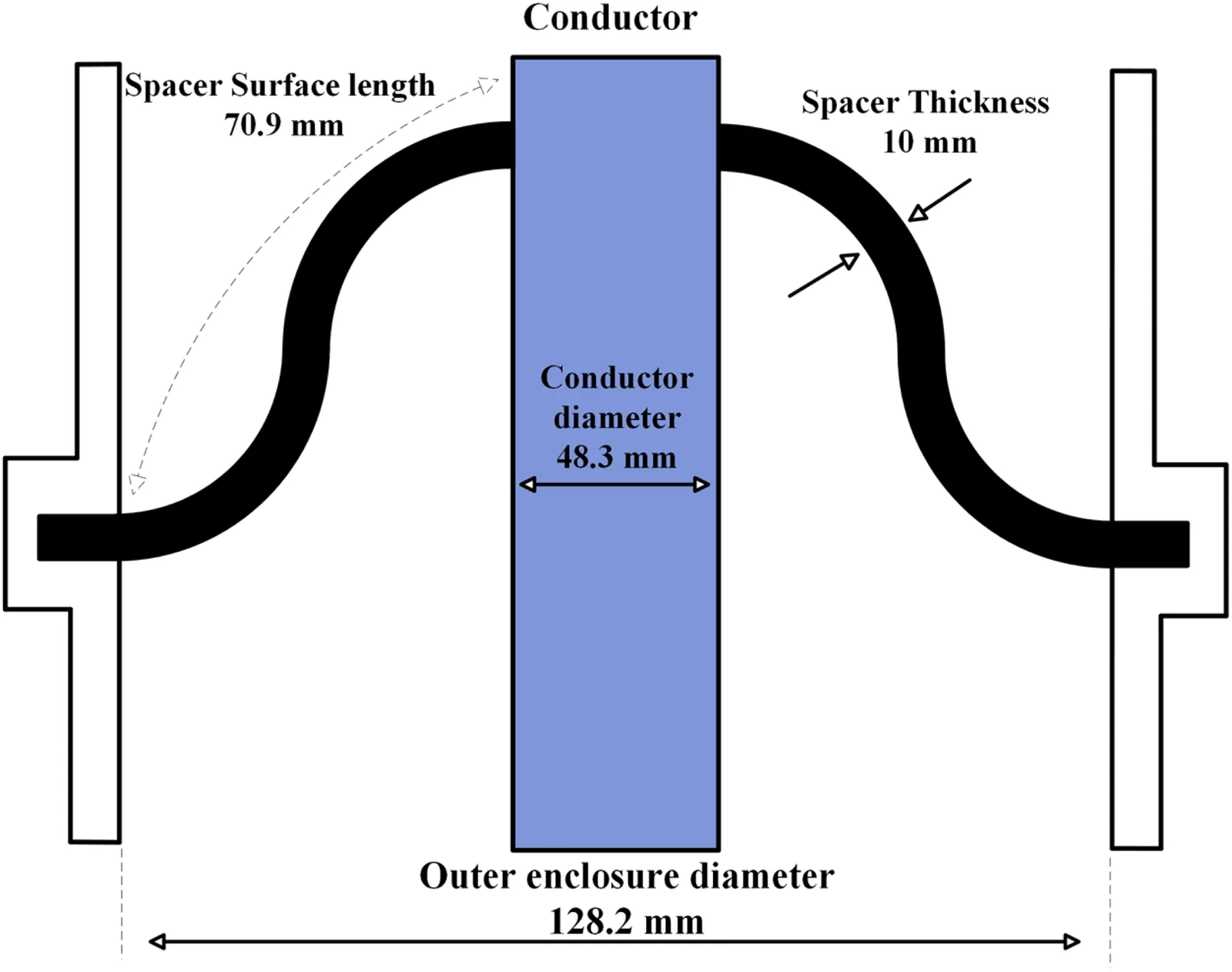
2D axisymmetric GIS spacer geometry in COMSOL adapted from ref. 27.

Coupled electrothermal simulation of an HVDC GIS spacer geometry was performed in COMSOL Multiphysics 6.3 using the geometry and DC operating conditions which were derived from a published HVDC spacer design.^33^ The model consists of a central conductor (48.3 mm outer diameter) energized at 131.5 kV DC, enclosed within a grounded cylindrical sheath (128.2 mm inner diameter), with the epoxy spacer occupying the annular insulation gap. A 2D axisymmetric representation was used, with the spacer arc length set to 70.9 mm as shown in Fig. 7. Thermal boundary conditions followed IEC 62271-1 for the worst-case operating scenario: 105 °C at the conductor and 70 °C at the enclosure boundary.

The bulk electrical conductivity of the epoxy under combined thermal and field stress was modelled using a thermally activated, field-enhanced conductivity expression:^34^11
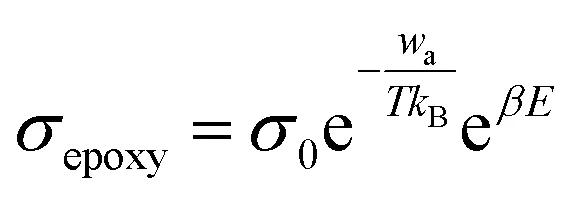
where *σ*_0_ is a conductivity constant (S m^−1^), *w*_a_ is the activation energy, *E* is the local electric field, and the field-dependence coefficient was fixed at *β* = 0.08 mm kV^−1^; *k*_B_ is the Boltzmann constant (eV K^−1^).^33^

For the reference epoxy system, a literature-based activation parameter of 0.95 eV was adopted.^34^ Because temperature-dependent conductivity was not measured separately for the individual formulations, formulation-dependent shifts in this parameter were estimated using the shallow relaxation time extracted from the ISPD fitting as a trap-informed surrogate. The estimation assumes that: (i) the effective thermally activated carrier-transport rate at the ISPD measurement temperature is inversely proportional to the shallow relaxation time, and (ii) the dominant transport mechanism and pre-exponential factors are comparable among the materials because the same epoxy matrix, filler loading, preparation procedure, and test conditions were employed. Under these assumptions, equating the relative relaxation rate to the corresponding Arrhenius transport factor gives:12
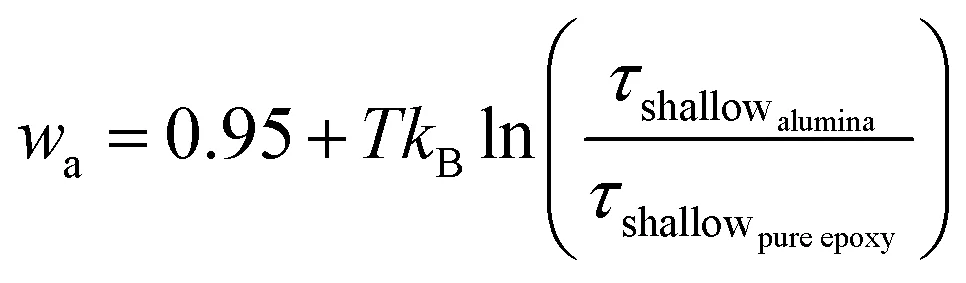


**Fig. 8:**
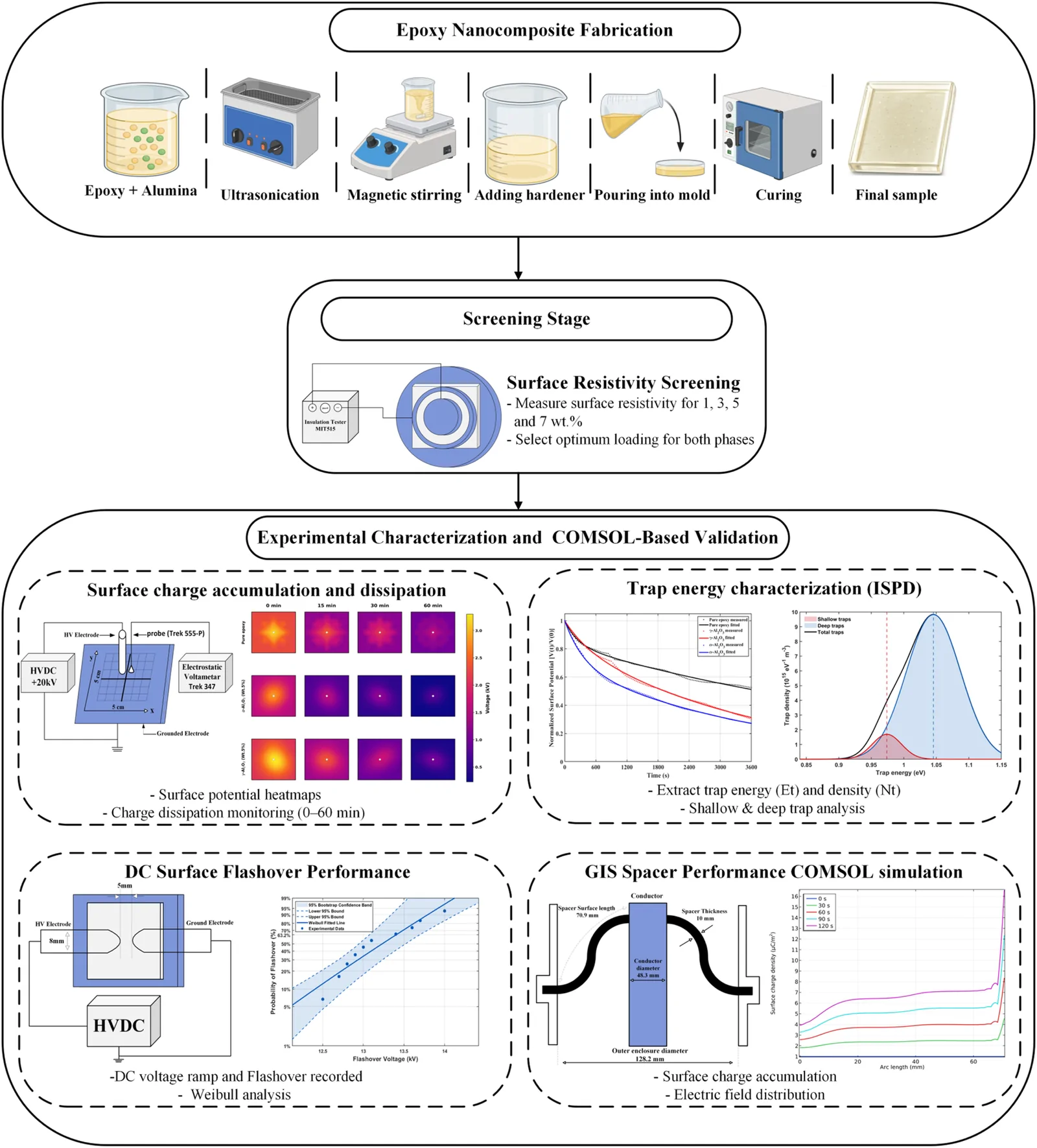
Schematic overview of the research methodology.

The overall experimental and numerical methodology adopted in this study is summarized in Fig. 8, starting from nanocomposite fabrication and optimization through surface resistivity screening, followed by multi-scale electrical characterization and GIS spacer performance validation using COMSOL Multiphysics.

## 3. Results and discussion

Results are presented following the multi-stage experimental framework described in Section 2. First, the phase identity and structural characteristics of the synthesized γ-rich alumina and α-Al_2_O_3_ nanofillers are established, providing the structural basis for interpreting their subsequent electrical behavior. Surface resistivity data across all concentrations are then used to identify the best performance loading for both phases, after which the best formulations are evaluated for surface charge dynamics, trap characteristics, and DC flashover performance. An integrated mechanistic interpretation then establishes the mechanistic links between these experimental observations. System-level validation through GIS spacer simulation concludes the section.

### 3.1 Characterization of alumina nanofillers

Phase identification and crystallinity quantification of the synthesized γ- and α-Al_2_O_3_ powders, performed as part of our companion study,^7^ are summarized here to establish the structural basis for the surface electrical behavior examined in the following sections.

**Fig. 9:**
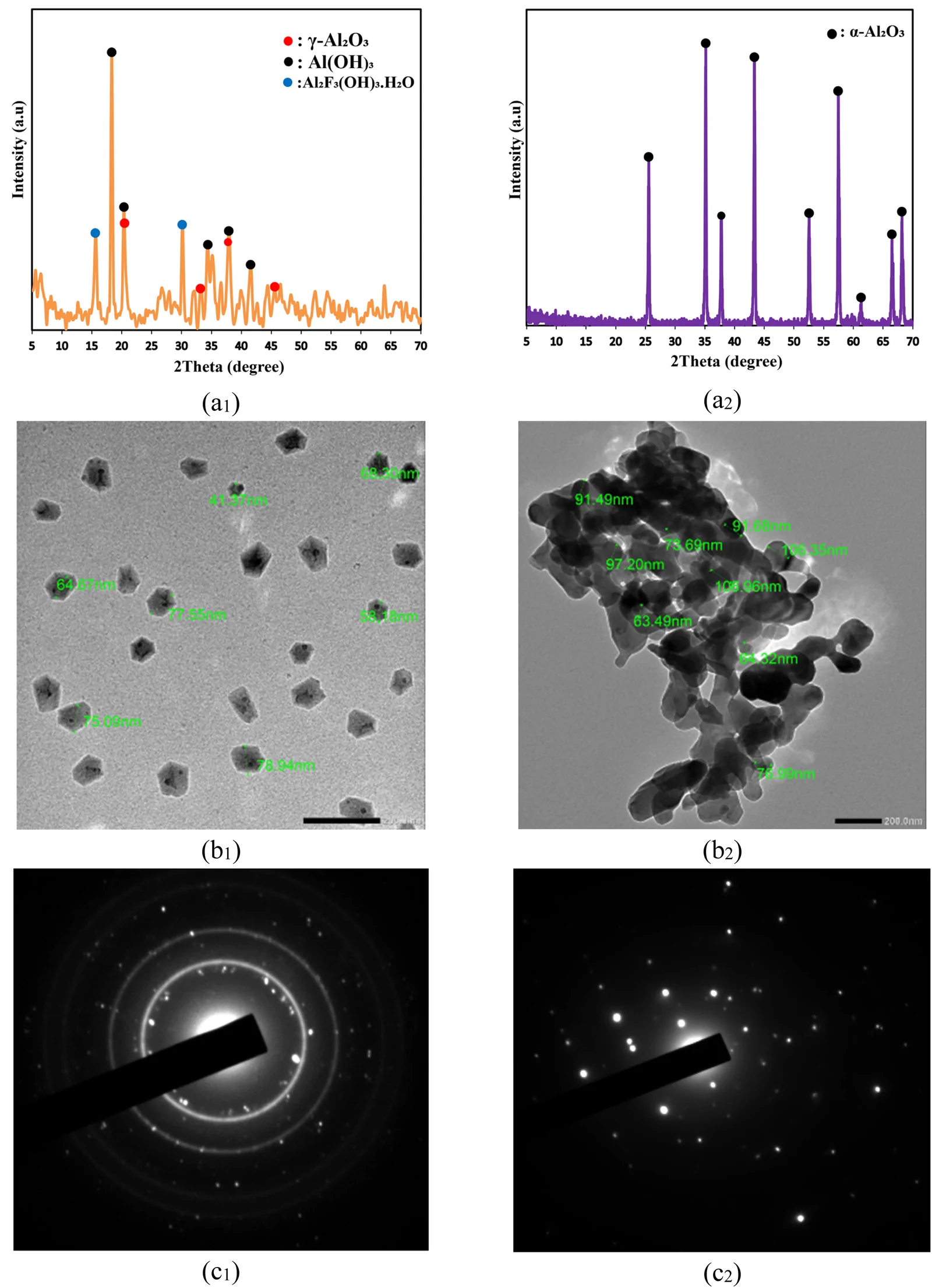
XRD pattern, HR-TEM images, SAED patterns of recycled alumina nanoparticles: (a_1_) XRD pattern (b_1_) HR-TEM image, (c_1_) SAED pattern of γ-rich alumina; (a_2_) XRD pattern (b_2_) HR-TEM image, (c_2_) SAED pattern of α-Al_2_O_3_ adapted from ref. 7.

The XRD pattern of the powder calcined at 600 °C, presented in Fig. 9a_1_, did not correspond to a single phase. Reflections at 2*θ* = 20.31°, 31.97°, 37.93°, and 45.69° were indexed to the (111), (220), (311), and (400) planes of cubic γ-Al_2_O_3_ (PDF#00-047-1292), while additional peaks confirmed the persistence of unconverted aluminum hydroxide in the form of anorthic gibbsite (PDF#096-101-1082) and monoclinic bayerite (PDF#96-100-0062). A minor reflection set was further assigned to trace aluminum fluoride hydroxide hydrate (Al_2_F_3_(OH)_3_·H_2_O, PDF#00-047-1784), One possible hypothesis is that this phase originated from fluoride-containing species associated with the surface treatment and/or coating system of the recycled cans; however, this attribution remains unverified because the original cans were not directly analyzed for fluorine. Quantitative phase analysis using Match! software (version 3.15) yielded an overall crystalline content of only 28.2% for this sample, reflecting the partial and incomplete nature of the dehydration–transformation process at 600 °C. the residual gibbsite, bayerite, and aluminum fluoride hydroxide hydrate phases to may influence dielectric properties through interfacial polarization, hydroxyl-related dipolar relaxation, and defect-assisted charge transport mechanisms, while the overall response is primarily associated with the γ-Al_2_O_3_-rich matrix.

Raising the calcination temperature to 1200 °C produced a markedly different diffraction profile, shown in Fig. 9a_2_, in which all reflections were indexed exclusively to α-Al_2_O_3_ (PDF#96-100-0033): 2*θ* = 25.57° (012), 35.19° (104), 37.38° (110), 43.25° (113), 52.43° (024), 57.39° (116), 61.21° (118), 66.43° (214), and 68.05° (300), corresponding to *d*-spacings of 3.4849, 2.5541, 2.3856, 2.0888, 1.7429, 1.6037, 1.5124, 1.4053, and 1.3763 Å, respectively. The absence of any residual hydroxide or transitional-phase reflections confirms that the reconstructive transformation to the corundum structure was complete at this temperature, and the corresponding calculated crystalline content rose sharply to 91.3%.^35–37^

HR-TEM imaging and the corresponding SAED patterns, presented in Fig. 9, provide independent confirmation of this structural contrast at the single-particle level. The γ-rich alumina sample shown in Fig. 9b_1_ and c_1_ displayed an irregular, pentagonal particle morphology; size measurements yielded diameters ranging from 33.1 to 95.2 nm with a mean of 70.3 nm. The associated SAED pattern consisted of diffuse, broadened rings rather than discrete spots, a signature of the poorly crystalline, multiphase character already identified by XRD. By contrast, the α-Al_2_O_3_ sample shown in Fig. 9b_2_ and c_2_ exhibited larger, more faceted particles, with diameters spanning 36.2 to 106.5 nm and a mean of 76.9 nm, consistent with grain growth and sintering at the higher calcination temperature. Its SAED pattern showed sharp, well-resolved diffraction spots, directly corroborating the single-phase, highly crystalline corundum structure indicated by XRD.^36–38^

Taken together, the XRD and HR-TEM/SAED results establish that the two alumina phases used in this study differ not only in crystal symmetry but in their overall structural order, defect content, and particle dimensions, differences that provide a direct physical rationale for the divergent trap energy distributions and surface resistivity behavior measured for the two nanofillers in the following sections.

**Fig. 10:**
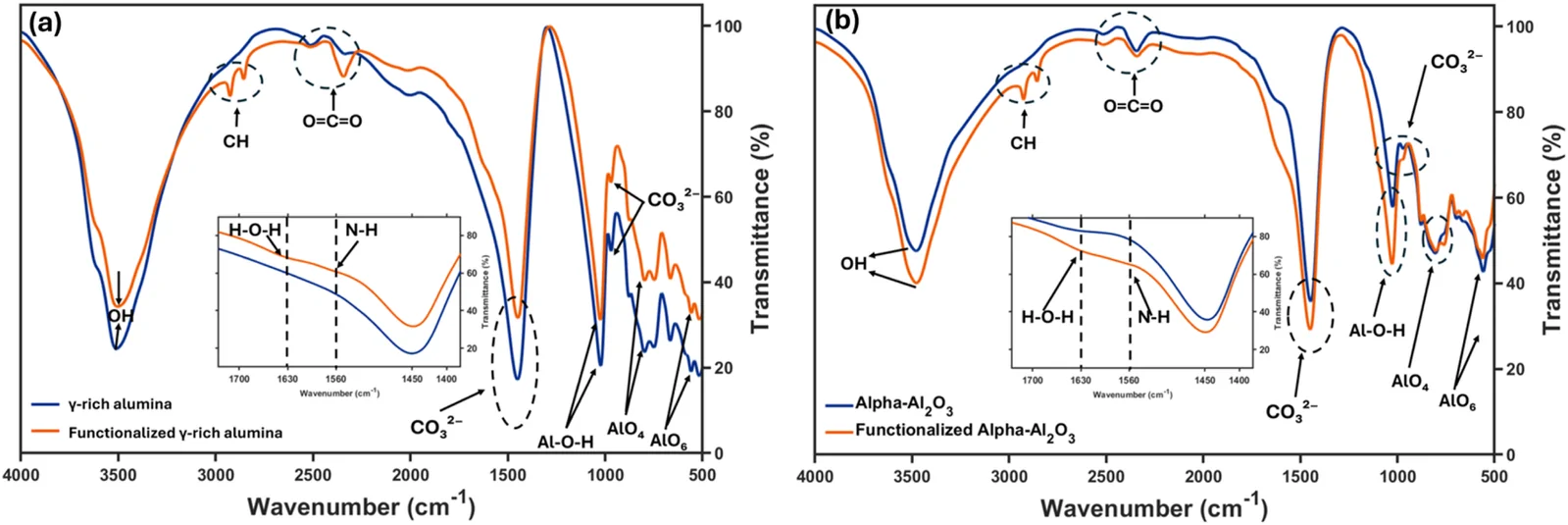
FTIR spectra of: (a) γ-rich recycled alumina, and (b) α-Al_2_O_3_ nanoparticles before and after APTES functionalization adapted from ref. 41.

Chemical changes induced by APTES treatment were evaluated by comparing the FTIR spectrum of each alumina nanofiller before and after functionalization, as presented in Fig. 10. Both γ-rich recycled alumina and α-Al_2_O_3_ exhibited new absorption bands within the 2800–3000 cm^−1^ region after treatment. In particular, the bands located at approximately 2925 and 2855 cm^−1^ are assigned to the asymmetric and symmetric stretching vibrations of the CH_2_ groups in the APTES propyl chain, respectively. The absence of these features from the untreated powders and their clear appearance following functionalization provide spectroscopic evidence for the retention of organosilane-derived groups on both alumina surfaces.^39^ The functionalized α-Al_2_O_3_ spectrum also displayed a band near 1560 cm^−1^, corresponding to the N–H bending vibration of the primary amine group. A distinct N–H band could not be resolved for the γ-rich recycled alumina because of its overlap with the strong carbonate-related absorption centred near 1450 cm^−1^ within the broader 1400–1650 cm^−1^ region.^40^ Functionalization additionally increased the absorption intensity between 1000 and 1130 cm^−1^. Since untreated alumina already exhibits Al–O–H contributions within this interval, only the treatment-induced increase is attributed to the formation of Si–O–Si and Al–O–Si linkages, supporting condensation and attachment of the silane species onto the oxide surface.^39^ Meanwhile, the Al–O lattice vibrations below 900 cm^−1^, associated with tetrahedrally and octahedrally coordinated aluminium, showed no appreciable shift after treatment. This preservation indicates that APTES functionalization altered the surface chemistry without measurably modifying the underlying alumina structure. Overall, the FTIR results provide complementary evidence for the successful functionalization of both nanofillers.

**Fig. 11:**
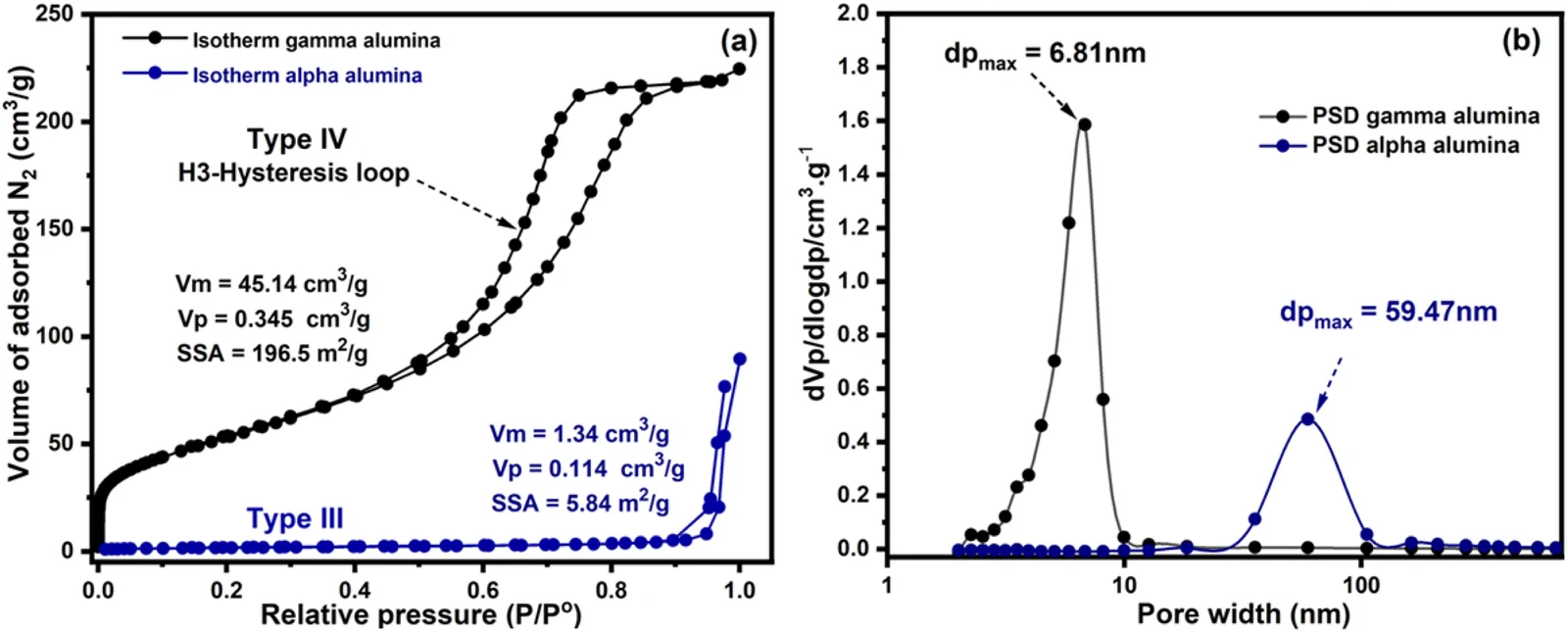
(a) N_2_-adsorption/desorption isotherms and (b) BJH-pore size distribution curve for alumina phases prepared from recycled aluminum can waste.

The nitrogen adsorption–desorption isotherms as presented in Fig. 11a, and pore size distributions shown in Fig. 11b clearly demonstrate the strong effect of calcination temperature on the textural evolution of alumina phases derived from Al-can waste. The alumina calcined at 600 °C exhibits a typical type IV isotherm with an H3 hysteresis loop, characteristic of mesoporous γ-rich Al_2_O_3_ composed of slit-like pores formed by aggregated plate-like particles. This sample shows a high BET specific surface area (≈196.5 m^2^ g^−1^), a large monolayer adsorption capacity (*V*_m_ ≈ 45.14 cm^3^ g^−1^) and a large total pore volume (≈0.345 cm^3^ g^−1^), with a narrow BJH-pore size distribution centered at ∼6.81 nm, confirming the dominance of uniform meso-porosity. In contrast, the alumina calcined at 1200 °C displays a type III isotherm, indicative of weak adsorbent–adsorbate interactions and a largely nonporous or macro-porous solid, consistent with the formation of thermodynamically stable α-Al_2_O_3_. This phase transformation is accompanied by a drastic reduction in surface area (≈5.84 m^2^ g^−1^), monolayer adsorption capacity (*V*_m_ ≈ 1.34 cm^3^ g^−1^) and pore volume (≈0.114 cm^3^ g^−1^), alongside a shift of the dominant pore diameter to much larger values (∼59.47 nm), attributed to sintering, grain growth, and collapse of the mesoporous framework at high temperature. These findings are completely matched with other previous work.^42–44^

### 3.2 Surface resistivity screening

Surface resistivity is one of the most important parameters governing charge transport along the gas–solid interface. A higher surface resistivity suppresses surface conduction, limits charge carrier mobility, and contributes to improved insulation performance under high-voltage DC operating conditions.^27,45^

Both alumina nanocomposite systems exhibited an improvement in surface resistivity relative to neat epoxy, with performance increasing with filler concentration up to a maximum at 5 wt%, as summarized in Table 1. Pure epoxy exhibited a surface resistivity of 2.13 × 10^12^ Ω per sq. γ-Rich alumina demonstrated a continuous enhancement across the full concentration range, reaching a maximum of 3.58 × 10^12^ Ω per sq at 5 wt%, the highest value recorded among all investigated compositions corresponding to an improvement of 68.1% relative to neat epoxy. Above 5 wt%, the surface resistivity decreased moderately to 3.14 × 10^12^ Ω per sq at 7 wt%, which may indicate the onset of particle agglomeration or interfacial saturation at higher loading levels.^6,7^ α-Al_2_O_3_ exhibited a more pronounced concentration threshold behavior, with resistivity remaining relatively modest at 1 and 3 wt% before rising sharply to 3.41 × 10^12^ Ω per sq at 5 wt%, representing an improvement of 60.1%. Above this concentration, the resistivity decreased more steeply to 2.46 × 10^12^ Ω per sq at 7 wt%, suggesting greater sensitivity to agglomeration compared with γ-rich alumina.

**Table 1:** Surface resistivity of pure epoxy and alumina-filled epoxy nanocomposites at 1, 3, 5, and 7 wt%

Material	Loading (wt%)	Surface resistivity mean ± SD (×10^12^ Ω per sq)	Improvement (%)
Pure epoxy	—	2.13 ± 0.07^a^	—
γ-Rich alumina	1	2.98 ± 0.09^c^	+39.9
	3	3.33 ± 0.11^e^	+56.3
	5	3.58 ± 0.12^f^	+68.1
	7	3.14 ± 0.10^d^	+47.4
α-Al_2_O_3_	1	2.52 ± 0.08^b^	+18.3
	3	2.54 ± 0.08^b^	+19.2
	5	3.41 ± 0.11^e^	+60.1
	7	2.46 ± 0.08^b^	+15.5

The enhancement in surface resistivity confirms that nanofiller incorporation effectively modifies the charge transport pathways along the epoxy surface. The polymer–filler interfacial regions introduced by nanoparticle incorporation act as barriers to charge carrier migration, increasing the resistance to surface conduction.^19,21^

A comparison between the two alumina phases reveals that γ-rich alumina outperformed α-Al_2_O_3_ across all concentrations. The superior surface resistivity of γ-rich alumina may be associated with its higher specific surface area (≈196.5 m^2^ g^−1^), which generates a larger total interfacial footprint per unit volume and introduces a greater density of charge-scattering sites along surface conduction pathways.^19^ Despite its lower specific surface area, α-Al_2_O_3_ still achieved a substantial 60.1% improvement at the 5 wt% loading, reflecting the effectiveness of the APTES-functionalized interfacial region in modifying charge transport regardless of the phase-specific surface area difference.^46,47^

Since surface resistivity directly influences charge migration and relaxation along the gas–solid interface, the differences observed between the two phases are expected to propagate into their surface charge accumulation and dissipation behavior, which is examined in the following section.

Surface-resistivity data for the nanocomposites were analyzed using a two-way ANOVA, with alumina phase (α and γ-rich) and filler loading (1, 3, 5, and 7 wt%) as fixed factors. Both main effects and the phase × loading interaction were statistically significant, as summarized in Table 2. Because the interaction was significant, the statistical interpretation focused primarily on comparisons among the phase–loading combinations using Tukey's HSD test at a significance level of 0.05. To include neat epoxy in the multiple comparisons presented in Table 1, a separate one-way ANOVA across all nine material formulations, followed by Tukey's HSD test, was performed. Means sharing at least one superscript letter are not significantly different, whereas means with no common letter differ significantly (*p* < 0.05). The results demonstrate that the effect of filler loading on surface resistivity is phase dependent. This behavior is consistent with differences in the dispersion and interfacial characteristics of the two alumina phases, rather than a common loading-dependent response.

**Table 2:** Two-way ANOVA results for the effects of alumina phase and filler loading on surface resistivity

Source	Degrees of freedom	*F*-Statistic	*p*-Value
Phase	1	567.97	<0.001
Loading	3	241.55	<0.001
Phase × loading	3	38.55	<0.001

**Fig. 12:**
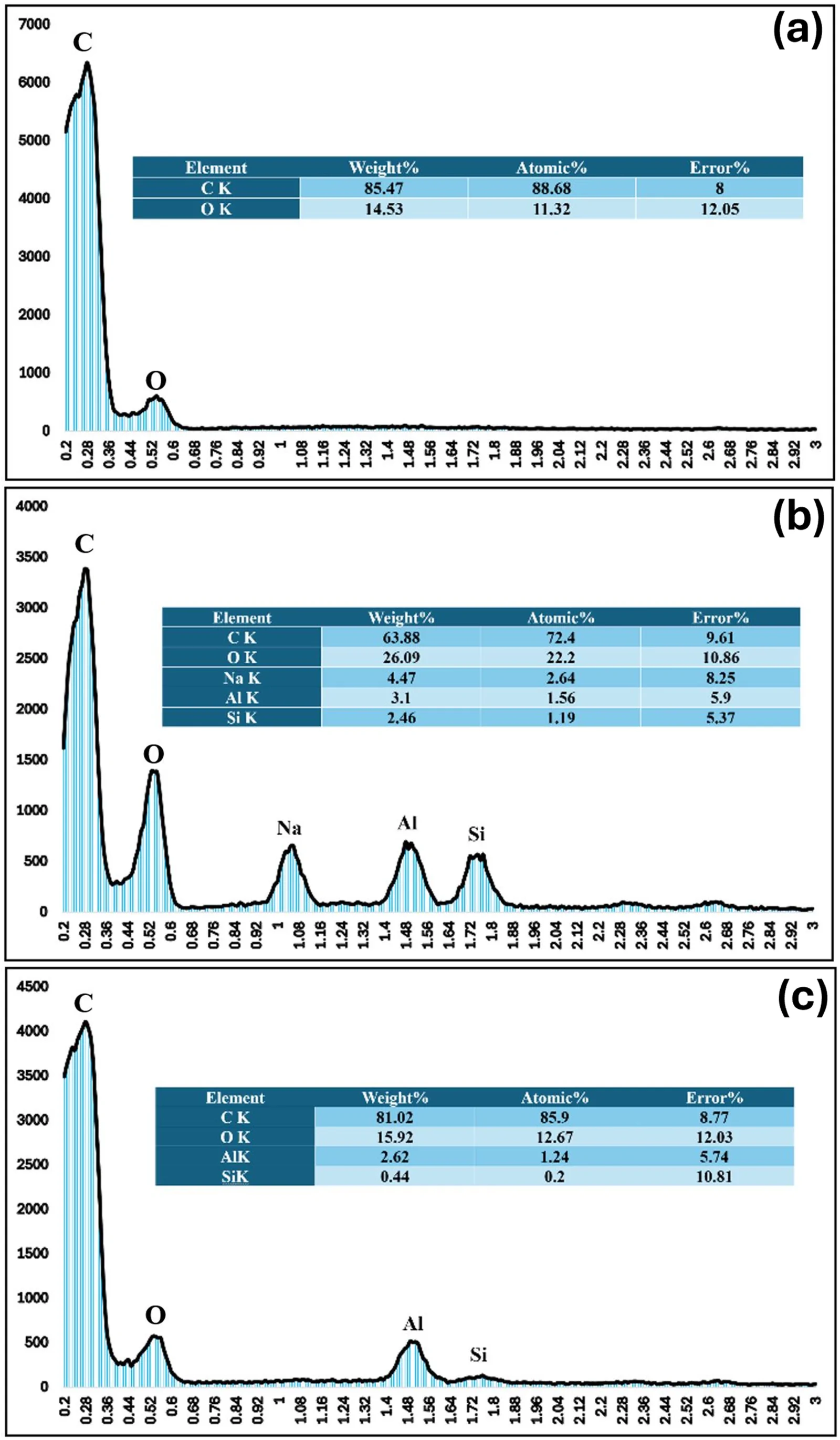
EDX spectra and corresponding elemental compositions of (a) neat epoxy, (b) γ-rich alumina/epoxy, and (c) α-Al_2_O_3_/epoxy composites adapted from ref. 7.

Energy-dispersive X-ray (EDX) spectroscopy was performed on the neat epoxy and on the γ-rich alumina/epoxy and α-Al_2_O_3_/epoxy composites at 5 wt% to verify filler incorporation and to assess the success of the APTES surface functionalization. The neat epoxy spectrum shown in Fig. 12a displays only the characteristic carbon and oxygen peaks of the polymer matrix, with carbon dominant (85.47 wt%) and no metallic or silicon signals, establishing a clean elemental baseline for comparison.

The γ-rich alumina/epoxy spectrum presented in Fig. 12b shows the characteristic Al and O peaks of the filler superimposed on the dominant C and O peaks of the epoxy, confirming successful incorporation of the alumina. A distinct Si peak at approximately 1.74 keV is also resolved, consistent with the grafting of silane moieties introduced during the APTES treatment. In addition, Na was quantitatively detected at 2.64 atomic% within the analyzed microregion. The sodium most likely originates from residual precursor-derived species associated with the Na_2_CO_3_ precipitation route. The α-Al_2_O_3_/epoxy spectrum presented in Fig. 12c likewise confirms the Al and O peaks of the filler, together with a weak Si signal at approximately 1.74 keV again consistent with APTES-derived silane grafting; no sodium is detected for this phase.

Residual sodium may nevertheless contribute to the measured electrical response. Mobile or weakly bound Na^+^ can increase ionic charge-carrier density and interfacial polarization, thereby influencing surface conductivity, charge accumulation, and charge relaxation. Previous controlled studies have demonstrated that Na^+^ incorporation can alter the epoxy crosslinked network and reduce electrical breakdown strength.^48^ However, the present results do not support mobile sodium as the dominant origin of the improved performance of the γ-rich composite. Despite containing detectable Na, this composite exhibited higher surface resistivity and slower early-stage potential decay than the α-Al_2_O_3_ composite, in which Na was below the EDX detection limit. These trends are opposite to those expected if enhanced ionic mobility were the controlling mechanism.

The contrast in silicon content between the two composites is itself informative. The markedly higher Si fraction on γ-rich alumina (≈2.5 wt%) relative to α-Al_2_O_3_ (≈0.4 wt%) is consistent with the substantially larger specific surface area of the defective-spinel γ phase, which presents a greater density of surface hydroxyl groups available for condensation with the APTES silanol groups, and therefore retains a higher grafted-silane coverage than the corundum-structured α phase.

The absence of a resolvable nitrogen peak does not contradict successful functionalization. It reflects the well-documented limitations of EDX for light elements, namely the low nitrogen concentration of the grafted amino silane and the strong absorption of the low-energy N-Kα signal (0.392 keV) within the alumina and polymer matrix.

**Fig. 13:**
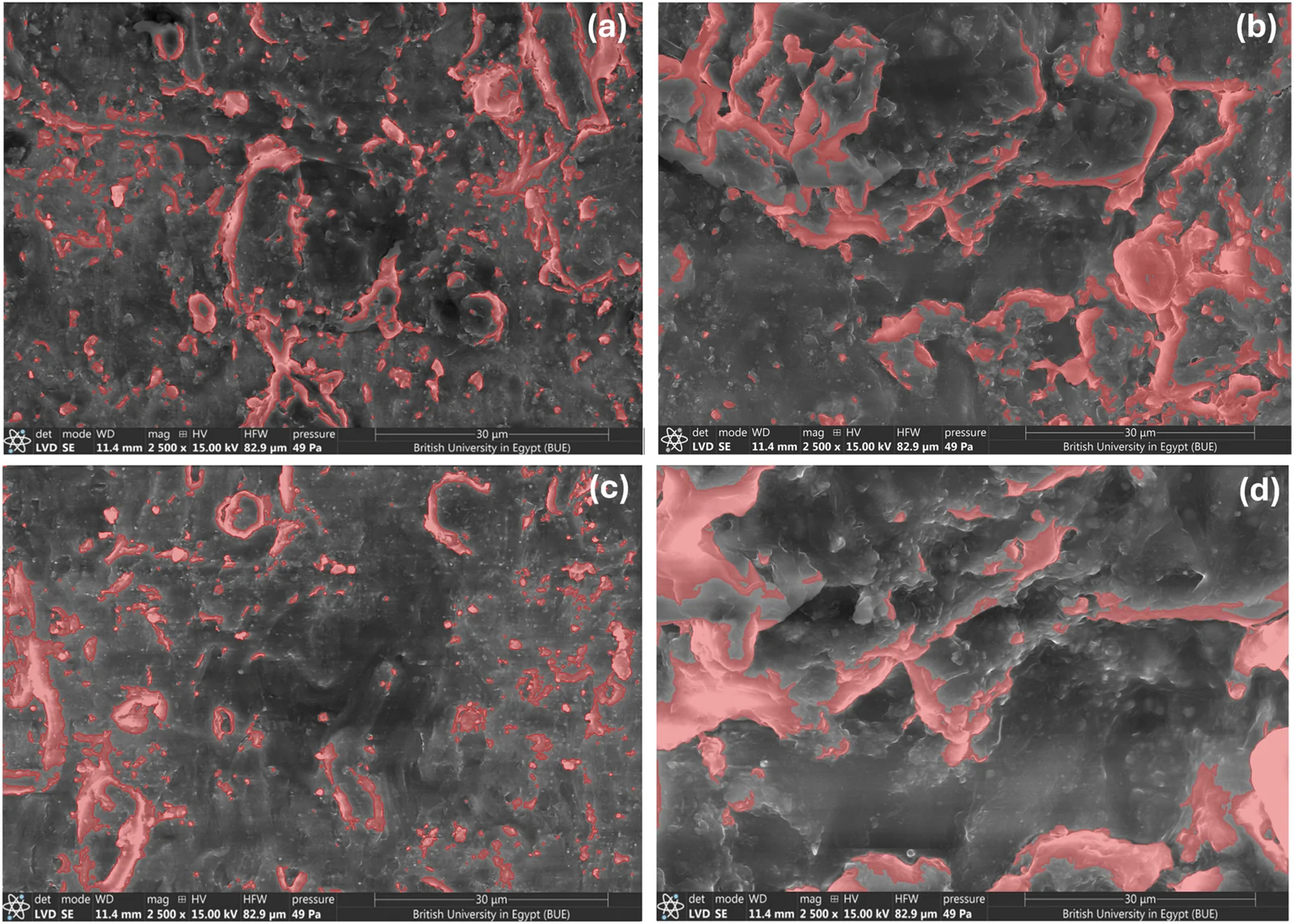
SEM micrographs with the segmented agglomerated regions highlighted in red: (a) γ-rich alumina/epoxy at 5 wt%, (b) γ-rich alumina/epoxy at 7 wt%, (c) α-Al_2_O_3_/epoxy at 5 wt%, and (d) α-Al_2_O_3_/epoxy at 7 wt% adapted from ref. 41.

The dispersion state of the alumina fillers within the epoxy matrix was examined by SEM for both phases at the 5 wt% loading and at the higher 7 wt% loading, in order to relate the observed degradation in electrical performance above 5 wt% to the underlying composite morphology. At 5 wt%, the γ-rich alumina/epoxy composite as shown in Fig. 13a, exhibits a relatively uniform distribution of finely dispersed filler particles across the fracture surface, with no extended voids and only limited surface relief. This homogeneous morphology promotes efficient interfacial contact between the alumina and the polymer and is consistent with the enhanced surface resistivity at this loading.

When the loading is increased to 7 wt%, the γ-rich alumina/epoxy composite presented in Fig. 13b, develops pronounced particle aggregates and coarse, raised clusters, indicating the onset of filler agglomeration. These aggregates introduce extended filler–filler contact regions and local heterogeneities that can act as sites of electric-field intensification and provide percolation-like pathways for charge transport, providing a direct morphological explanation for the deterioration in surface insulation performance observed beyond the optimal loading.

The α-Al_2_O_3_/epoxy composite follows the same trend. At 5 wt% presented in Fig. 13c the surface is comparatively smooth, with fine α-Al_2_O_3_ particles homogeneously distributed and free of large agglomerates or interconnected clusters, reflecting good wetting and dispersion of the filler. At 7 wt% shown in Fig. 13d, the morphology becomes markedly more heterogeneous, with visible particle clusters and an isolated cavity region, again signifying agglomeration at the elevated loading.

To complement the qualitative SEM observations, a semi-quantitative image analysis was performed on micrographs acquired at the same magnification (2500×) and processed using an identical segmentation procedure.^49,50^ Following automatic Otsu thresholding, connected high-contrast domains with an equivalent diameter ≥ 0.5 µm were classified as apparent agglomerated regions. Their area fraction, number density, mean equivalent diameter, and D90 equivalent diameter are summarized in Table 3. Increasing the filler loading from 5 to 7 wt% increased the agglomerated area fraction from 14.1% to 22.5% for γ-rich alumina and from 15.0% to 21.7% for α-Al_2_O_3_. The corresponding D90 values increased from 2.70 to 3.67 µm and from 3.11 to 4.62 µm, respectively, whereas the agglomerate density decreased. These combined changes indicate the coalescence of smaller dispersed domains into fewer and larger agglomerates at 7 wt%.

**Table 3:** Semi-quantitative agglomeration descriptors extracted from SEM micrographs adapted from ref. 41

Parameter	γ-Rich alumina 5 wt%	γ-Rich alumina 7 wt%	α-Al_2_O_3_ 5 wt%	α-Al_2_O_3_ 7 wt%
Agglomerated area fraction (%)	14.1	22.5	15.0	21.7
Agglomerate density (per 1000 µm^2^)	30.9	20.4	27.8	12.7
Mean equivalent diameter (µm)	1.64	2.17	1.77	2.33
D90 equivalent diameter (µm)	2.70	3.67	3.11	4.62

### 3.3 Surface potential distribution and decay

The accumulation and dissipation of surface charges play a critical role in determining the insulation reliability of polymeric materials operating under high electric stress. Evaluating the surface potential decay behavior therefore provides valuable insight into the capacity of the developed nanocomposites to suppress charge accumulation and facilitate charge relaxation along the gas–solid interface.^51–53^

The peak surface potential results reveal distinct differences in charge dissipation behavior among the investigated materials over the 60 minute decay period, as summarized in Table 4. Pure epoxy exhibited an initial peak potential of 3.20 kV, which decreased gradually to 1.66 kV after 60 minutes, corresponding to a peak surface potential decay ratio of 48.0%. Both alumina nanocomposites demonstrated substantially enhanced charge dissipation performance relative to the unfilled matrix. γ-Rich alumina reduced the peak surface potential from 3.30 kV to 1.00 kV, yielding a dissipation ratio of 69.7%, while α-Al_2_O_3_ reached a residual surface potential of 0.77 kV after 60 minutes, corresponding to the highest dissipation ratio of 72.7% among the alumina-based systems.

**Table 4:** Peak surface potential values of pure epoxy and optimized alumina nanocomposites during the charge dissipation process (0–60 min)

Material	0 min (kV)	15 min (kV)	30 min (kV)	60 min (kV)
Pure epoxy	3.20	2.40	2.08	1.66
γ-Rich alumina (5 wt%)	3.30	2.35	1.72	1.00
α-Al_2_O_3_ (5 wt%)	2.82	1.58	1.24	0.77

**Fig. 14:**
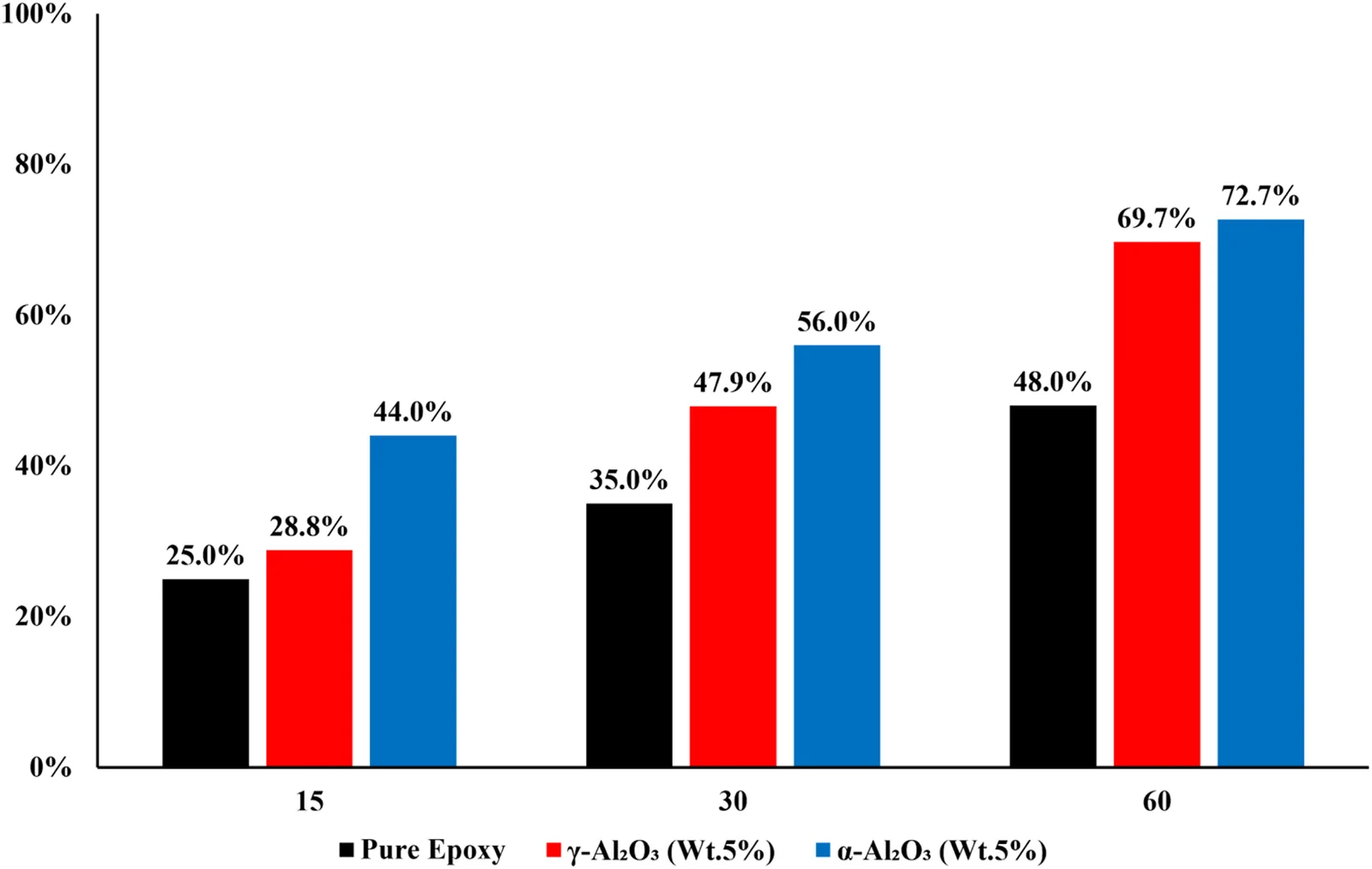
Surface potential reduction percentage of pure epoxy and alumina-filled epoxy nanocomposites as a function of charge decay time.

The temporal evolution of the dissipation ratios further reveals differences in the rate of charge relaxation between the two phases as shown in Fig. 14. After 15 minutes, α-Al_2_O_3_ exhibited the highest dissipation ratio of 44.0%, substantially exceeding γ-rich alumina at 28.8% and pure epoxy at 25.0%, indicating that α-Al_2_O_3_ promotes markedly faster early-stage charge release. As the decay process progressed to 60 minutes, γ-rich alumina narrowed the gap considerably, reaching 69.7% compared with 72.7% for α-Al_2_O_3_, while pure epoxy remained the lowest at 48.0%. This temporal reversal in relative performance with α-Al_2_O_3_ leading at early times and both phases converging at 60 minutes suggests that the two phases differ not only in the total quantity of trapping sites but also in their energy depth, a distinction that is examined directly through the ISPD trap characterization presented in Section 3.4.

**Fig. 15:**
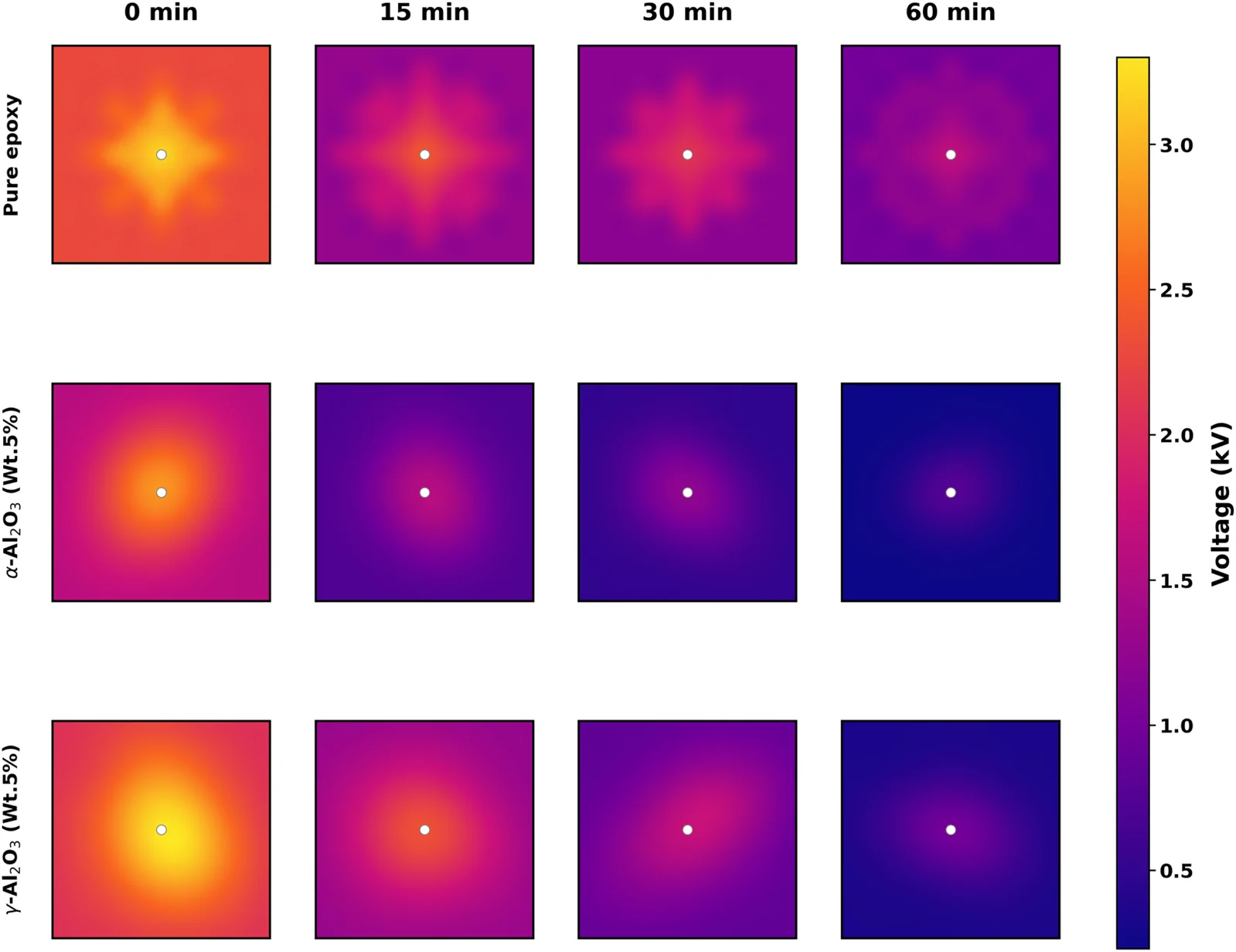
Evolution of surface potential distributions for pure epoxy and alumina-filled epoxy nanocomposites during charge dissipation at 0, 15, 30, and 60 min after DC charging.

The surface potential distribution maps presented in Fig. 15 provide further visual confirmation of the enhanced charge dissipation behavior across the decay period. At the beginning of the decay process, all materials exhibited localized regions of high surface potential concentrated around the charging point. With increasing decay time, the intensity of these regions decreased progressively, accompanied by a reduction in the spatial extent of the charged area. Compared with pure epoxy, both alumina nanocomposites exhibited a faster reduction in surface potential magnitude and charged area, particularly after 30 and 60 minutes. The maps corresponding to α-Al_2_O_3_ show a more rapid transition toward lower potential levels in the early stages of decay, while γ-rich alumina demonstrated a more progressive but ultimately comparable charge relaxation over the full 60 minute period. Pure epoxy retained a relatively broader high-potential region throughout the decay window, reflecting its weaker capacity to dissipate accumulated surface charge.

### 3.4 Trap energy characterization

Trap characteristics provide valuable insight into the charge transport and relaxation processes governing the behavior of polymeric insulation systems.^54,55^ Since surface charge accumulation and dissipation are strongly influenced by charge trapping and de-trapping mechanisms, analysis of the trap energy distribution offers a physical explanation for the differences in charge dissipation behavior observed among the investigated materials in Section 3.3.

**Fig. 16:**
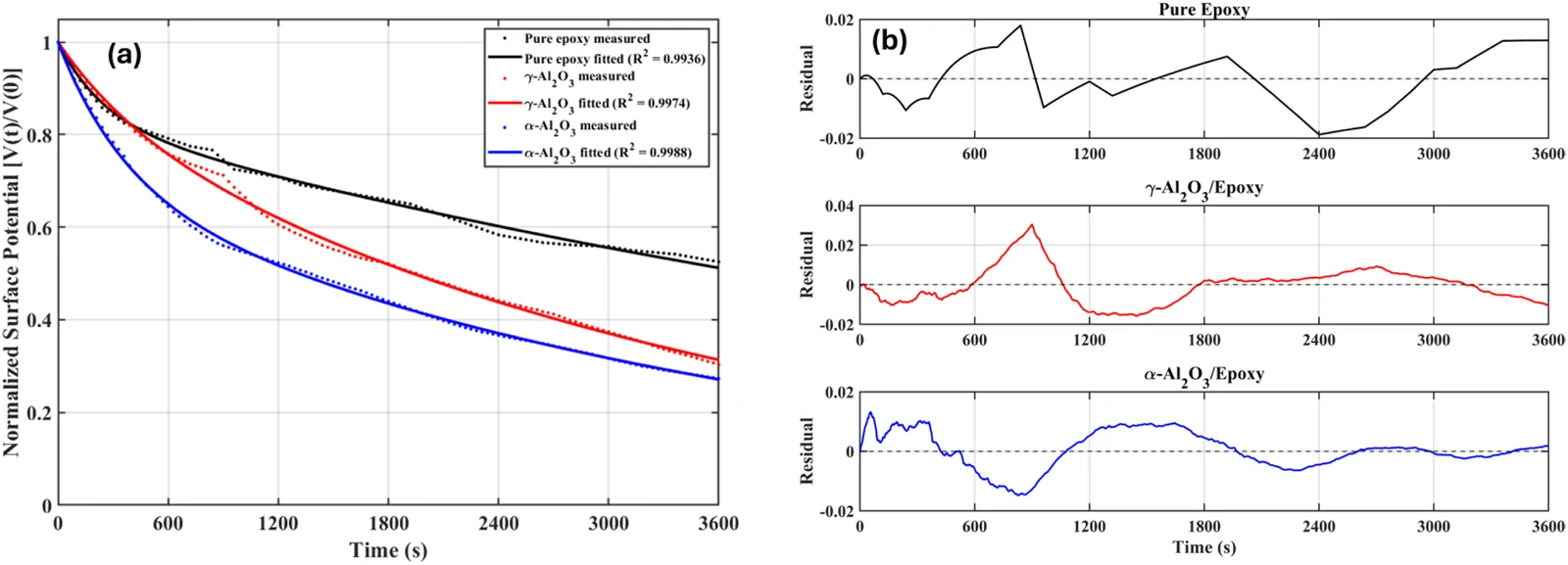
(a) Normalized surface potential decay, and (b) residuals between the measured normalized surface potential decay data and the corresponding dual-exponential fits for neat epoxy, γ-rich Al_2_O_3_/epoxy, and α-Al_2_O_3_/epoxy.

The normalized surface potential decay curves obtained from ISPD analysis reveal clear differences in charge relaxation kinetics between the two alumina phases and the unfilled epoxy as shown in Fig. 16a. The residuals remained small relative to the overall normalized-potential range as shown in Fig. 16b, although modest time-dependent deviations were observed. Together with the high values (*R*^2^ > 0.9936), these results indicate that the dual-exponential function provides a very good phenomenological representation of the overall relaxation behavio. Pure epoxy exhibited the slowest decay throughout the entire 3600 seconds measurement period, retaining more than half of its initial normalized surface potential at the end of the decay window. Both alumina nanocomposites demonstrated substantially faster potential dissipation, confirming that nanofiller incorporation meaningfully altered the charge transport characteristics of the epoxy matrix. Between the two phases, α-Al_2_O_3_ exhibited the more rapid early decay, while both phases converged toward comparable dissipation levels over longer timescales.

**Fig. 17:**
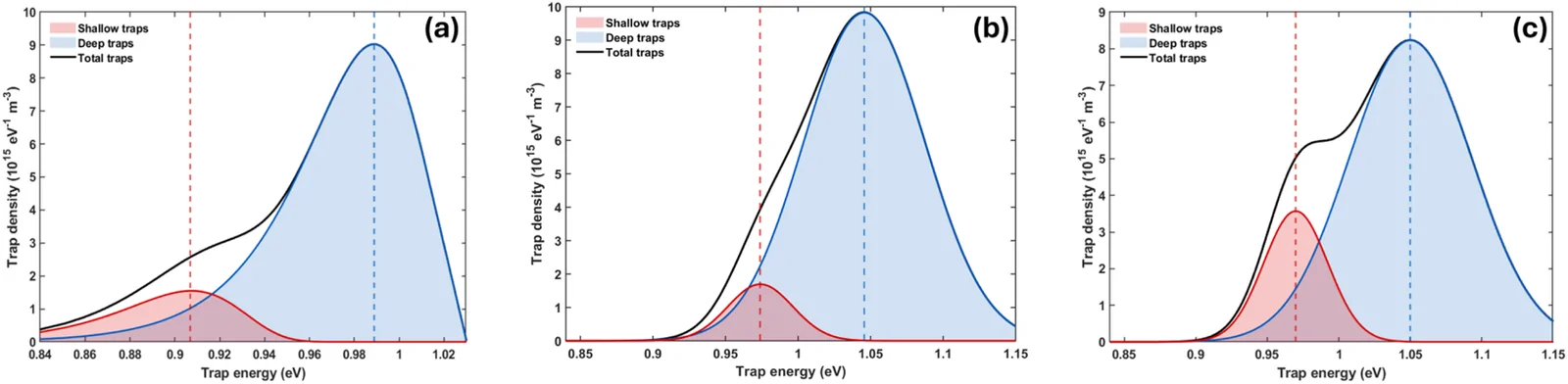
Trap energy distributions of (a) pure epoxy, (b) γ-rich alumina, and (c) α-Al_2_O_3_.

The extracted trap parameters are summarized in Table 5 and Fig. 17. Pure epoxy exhibited shallow and deep trap energies of 0.9069 and 0.9888 eV, respectively. Following nanofiller incorporation, both trap energies shifted toward higher values for both alumina phases. The shallow trap energy increased to 0.9738 eV for γ-rich alumina and 0.9700 eV for α-Al_2_O_3_, while the deep trap energy increased more substantially to 1.0456 eV for γ-rich alumina and 1.0499 eV for α-Al_2_O_3_, indicating that the APTES-functionalized alumina surfaces introduce more energetically stable trapping states into the epoxy matrix through the formation of additional polymer–filler interfacial regions.

**Table 5:** Shallow and deep trap parameters extracted by ISPD analysis for pure epoxy and the selected 5 wt% alumina nanocomposites

Material	Shallow trap energy (eV)	Deep trap energy (eV)	Max shallow density (×10^15^ eV^−1^ m^−3^)	Max deep density (×10^15^ eV^−1^ m^−3^)
Pure epoxy	0.9069	0.9888	1.5493	9.0305
γ-Rich alumina (5 wt%)	0.9738	1.0456	1.6925	9.8191
α-Al_2_O_3_ (5 wt%)	0.9700	1.0499	3.5711	8.2299

The trap parameters provide a direct explanation for the charge dissipation behavior described in Section 3.3. During the initial decay stage, particularly within the first 15 minutes, α-Al_2_O_3_ exhibited the highest dissipation ratio of 44.0%, substantially exceeding γ-rich alumina at 28.8%. This trend corresponds directly to the shallow trap density distribution: α-Al_2_O_3_ possesses the highest shallow trap density among all investigated materials (3.5711 × 10^15^ eV^−1^ m^−3^), more than double that of γ-rich alumina (1.6925 × 10^15^ eV^−1^ m^−3^). Since shallow traps are characterized by comparatively low trapping energies, carriers captured at these states can be released more readily at ambient temperature, producing the faster initial charge relaxation observed for α-Al_2_O_3_.

The long-term dissipation behavior after 60 minutes follows a different logic. α-Al_2_O_3_ achieved a dissipation ratio of 72.7%, marginally exceeding γ-rich alumina at 69.7%. Despite the slightly deeper trap energy of α-Al_2_O_3_ (1.0499 eV *vs.* 1.0456 eV for γ), its lower deep trap density (8.2299 × 10^15^ eV^−1^ m^−3^*vs.* 9.8191 × 10^15^ eV^−1^ m^−3^ for γ) means that fewer carriers are immobilized in long-lived deep trapping states over the 60 min observation window. Consequently, α-Al_2_O_3_ exhibited a slightly lower residual peak surface potential after 60 minutes than the γ-rich alumina composite. These results confirm that long-term charge dissipation is governed by the combined contribution of deep trap density and trap energy depth rather than by either parameter independently.

Taken together, these observations indicate that the two alumina phases exhibit different effective trap landscapes. The higher effective deep-trap density of the γ-rich alumina composite immobilizes mobile carriers and suppresses their contribution to surface conduction, consistent with its higher surface resistivity. However, this carrier immobilization does not constitute charge removal; the longer residence time associated with deep states may also contribute to residual charge retention and slower post-stress potential decay. Conversely, the greater effective shallow-trap contribution of the α-Al_2_O_3_ composite facilitates faster de-trapping, carrier migration, and subsequent neutralization, consistent with its more rapid early-stage surface-potential decay. Thus, suppression of mobile charge transport during electrical stressing and relaxation of residual surface potential after voltage removal are physically distinct, although interconnected, processes.

Since surface-charge dynamics influence the local electric-field distribution at the gas–solid interface, the differences in surface resistivity and surface-potential decay observed between the two phases may also influence their surface-flashover performance.

### 3.5 DC surface flashover performance

Surface flashover voltage is one of the most critical indicators of insulation reliability in polymeric GIS spacer systems. Since flashover propagation occurs along the spacer surface, its behavior is directly influenced by surface charge transport processes, charge accumulation characteristics, and the capacity of the material to suppress local electric-field enhancement.^56^

Both alumina nanocomposites exhibited improved flashover performance relative to pure epoxy, as summarized in Table 6. The mean flashover voltage increased from 11.96 kV for pure epoxy to 13.49 kV for γ-rich alumina and 13.17 kV for α-Al_2_O_3_, corresponding to numerical improvements of 12.8% and 10.1%, respectively. γ-Rich alumina achieved the highest numerical mean flashover voltage among all investigated compositions in this study.

**Table 6:** Statistical and Weibull parameters of DC surface flashover characteristics for pure epoxy and optimized alumina nanocomposites

Material	No. of readings	Mean flashover voltage (kV)	Std deviation (kV)	*α* (kV)	*β*	*R* ^2^	95% CI		
							Mean (kV)	*α* (kV)	*β*
Pure epoxy	10	11.96	0.502	12.2	25.32	0.901	11.60–12.32	11.86–12.49	19.82–41.28
γ-Rich alumina (5 wt%)	10	13.49	0.65	13.8	22.19	0.925	13.02–13.96	13.39–14.15	17.17–33.61
α-Al_2_O_3_ (5 wt%)	10	13.17	0.49	13.4	29.07	0.914	12.82–13.52	13.07–13.69	22.25–46.38

Although the γ-rich composite exhibited the highest numerical mean, a direct comparison using Welch's independent-samples *t*-test showed that its difference from the α-Al_2_O_3_ composite was not statistically significant (*p*-value = 0.23 > 0.05). Therefore, the two nanocomposites exhibited statistically comparable flashover performance under the present experimental condition.

**Fig. 18:**
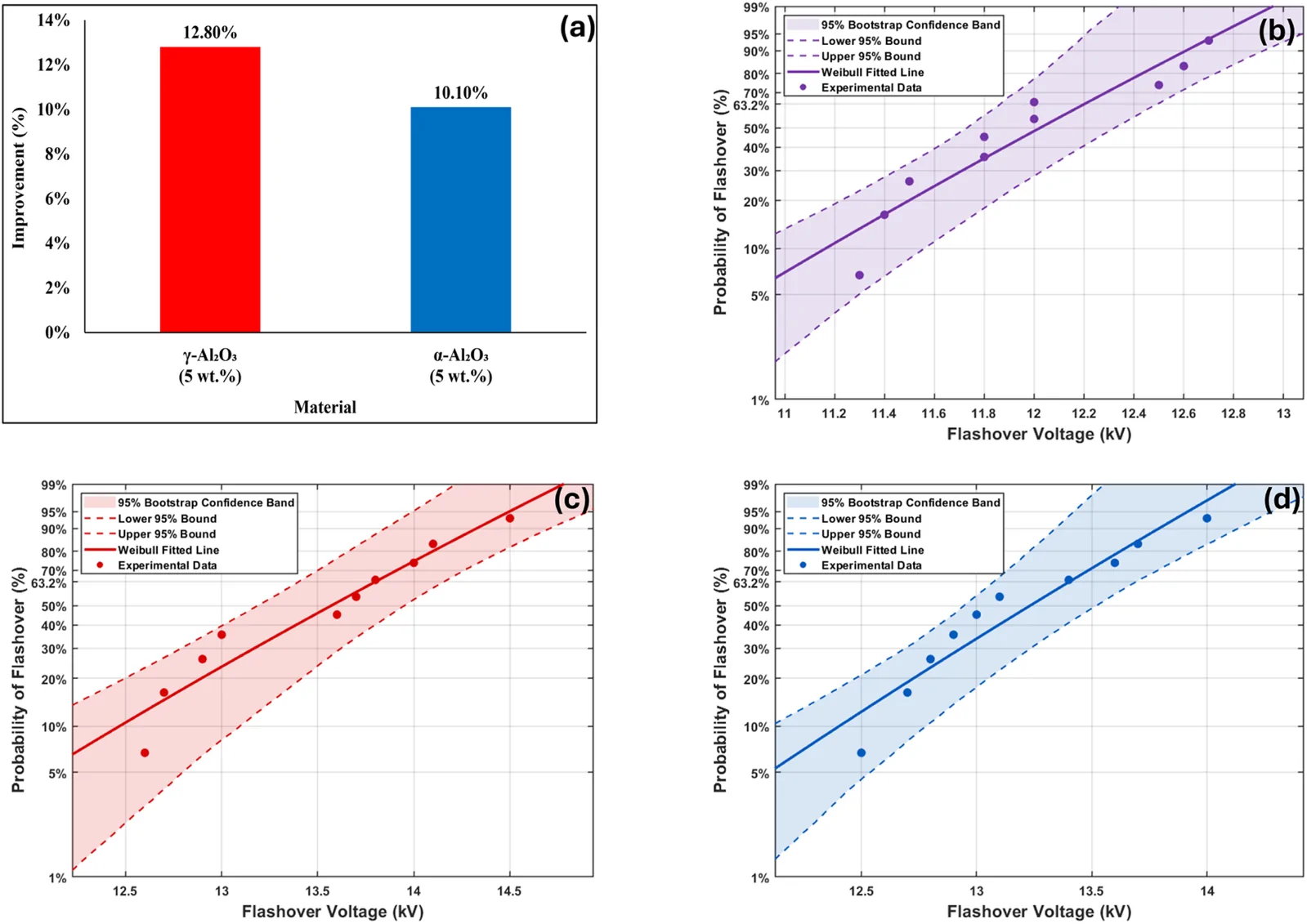
(a) Percentage of improvement, and Weibull distribution of DC surface flashover voltage with 95% bootstrap confidence bounds for: (b) pure epoxy, (c) γ-rich alumina, and (d) α-Al_2_O_3_.

The Weibull statistical analysis confirms the reliability of these results. The characteristic flashover voltage (*α*) increased from 12.2 kV for pure epoxy to 13.8 kV for γ-rich alumina and 13.4 kV for α-Al_2_O_3_. The shape parameter *β* provides additional insight into measurement consistency: α-Al_2_O_3_ exhibited the highest *β* value of 29.07, indicating the lowest data scatter and most predictable flashover behavior among the three materials, while γ-rich alumina yielded a *β* of 22.19, reflecting greater variability that may be associated with the more heterogeneous surface structure introduced by its high-specific-surface-area morphology. The coefficient of determination *R*^2^ exceeded 0.90 for all materials, confirming satisfactory Weibull fit quality. The bootstrap confidence intervals quantify the uncertainty associated with the estimated Weibull parameters and mean flashover voltages. While both nanocomposites exhibited higher mean and characteristic flashover voltages than pure epoxy, the confidence intervals of the γ-rich and α-Al_2_O_3_ composites overlapped. Consistent with the direct Welch's *t*-test, the phase-to-phase difference was not statistically significant as shown in Fig. 18.

### 3.6 Integrated mechanistic interpretation of surface resistivity, charge dynamics, and flashover performance

This section provides an integrated mechanistic interpretation of the surface-resistivity, charge-dissipation, trap-characterization, and flashover results. It is intended to identify qualitative physical relationships among the measured properties rather than to establish a formal statistical correlation.

**Fig. 19:**
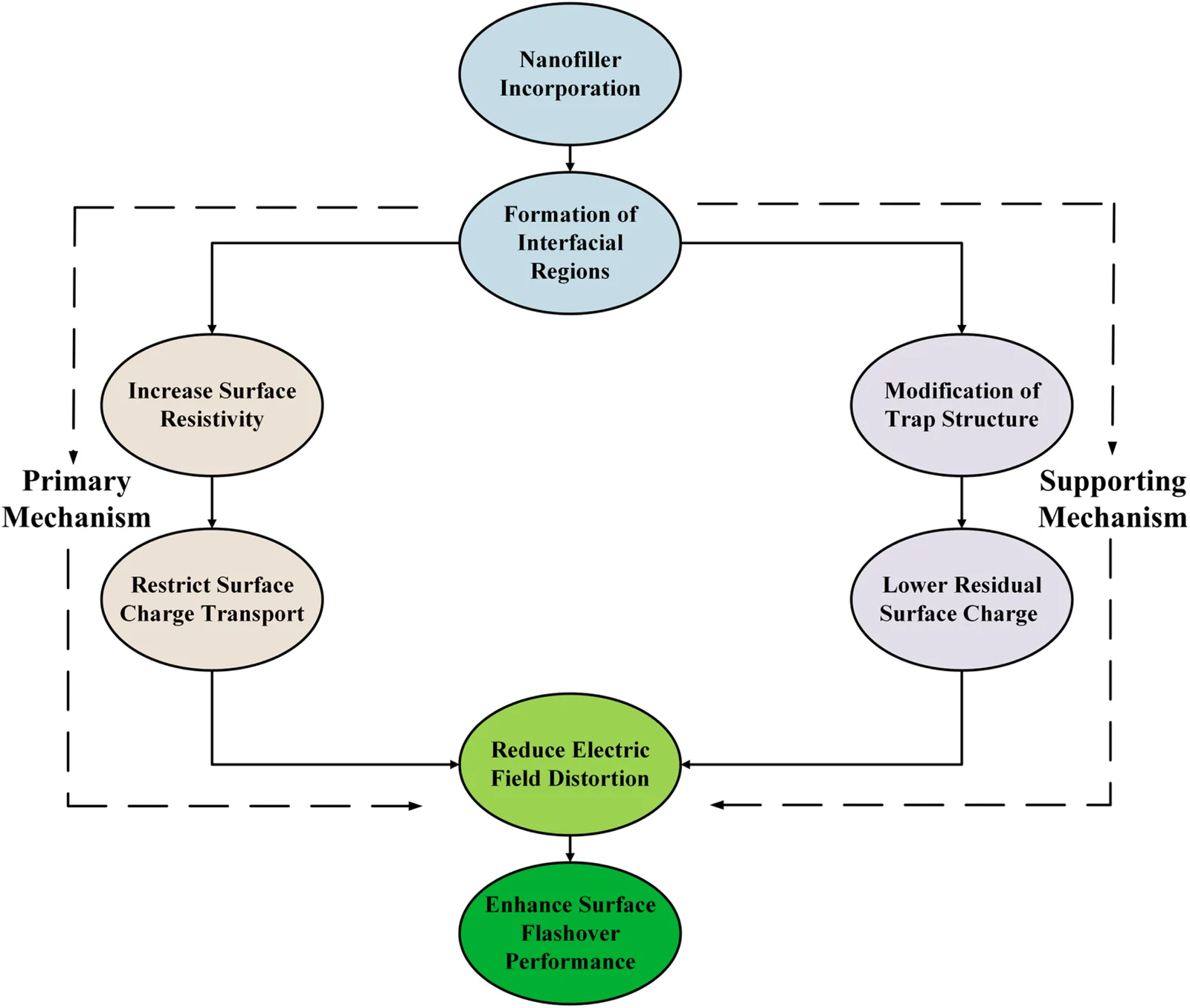
Proposed enhancement mechanism illustrating the influence of nanofiller incorporation on surface charge dynamics and flashover performance of epoxy nanocomposites.

A particularly revealing observation emerges when comparing the flashover results with the charge dissipation behavior. Although the flashover-voltage numerical ranking (γ-rich alumina > α-Al_2_O_3_ > pure epoxy) precisely reproduces the surface-resistivity ranking, it does not follow the charge-dissipation ranking, in which α-Al_2_O_3_ consistently outperformed γ-rich alumina at both early and late stages of the decay process. This divergence is not a contradiction; rather, it points to the simultaneous operation of two complementary mechanisms, each acting on the flashover performance through a different pathway and on a different timescale as shown in Fig. 19.

The first and dominant mechanism under the present testing conditions is surface resistivity. The exact one-to-one correspondence between resistivity and the numerical flashover rankings strongly suggests that suppressing surface charge transport is the dominant factor under the present pre-discharged testing conditions controlling flashover initiation. Higher surface resistivity limits the mobility of charge carriers along the gas–solid interface, reduces the formation of localized conductive pathways, and consequently constrains the field enhancement that drives streamer propagation. Since the flashover tests in this work were performed on pre-discharged specimens, the accumulated charge history prior to each test was minimal, placing surface resistivity in its position of maximum influence.

The second mechanism is trap-mediated charge dissipation which operates in parallel but is not fully expressed under these laboratory conditions. Faster charge relaxation reduces the residual surface charge between stress events and limits the charge available to sustain field distortion. Under the controlled pre-discharge protocol adopted here, this contribution is secondary. However, under realistic HVDC service conditions, where charge accumulates continuously along the spacer surface over time without systematic discharge, the relative importance of this mechanism would increase substantially. In such pre-charged configurations, the superior early-stage charge relaxation capability of α-Al_2_O_3_ rooted in its elevated shallow trap density would be expected to play a progressively more influential role, potentially narrowing the performance gap; this should be verified in future pre-charged flashover experiments under prolonged DC stress.

The overall picture is therefore one of complementarity rather than conflict: surface resistivity and trap-mediated charge dissipation act together on the flashover performance, with the former dominating under clean-surface laboratory testing and the latter gaining relative importance as operating conditions approach the sustained charge accumulation scenario of long-term HVDC service.

### 3.7 GIS spacer performance under electrothermal DC stress

Material-level improvements in surface resistivity and charge dissipation are meaningful only if they translate into reduced charge accumulation and suppressed field distortion at the system level. Coupled electrothermal simulation of the GIS spacer geometry was therefore conducted to assess whether the phase-dependent performance differences identified experimentally manifest under representative electrothermal DC conditions.

**Fig. 20:**
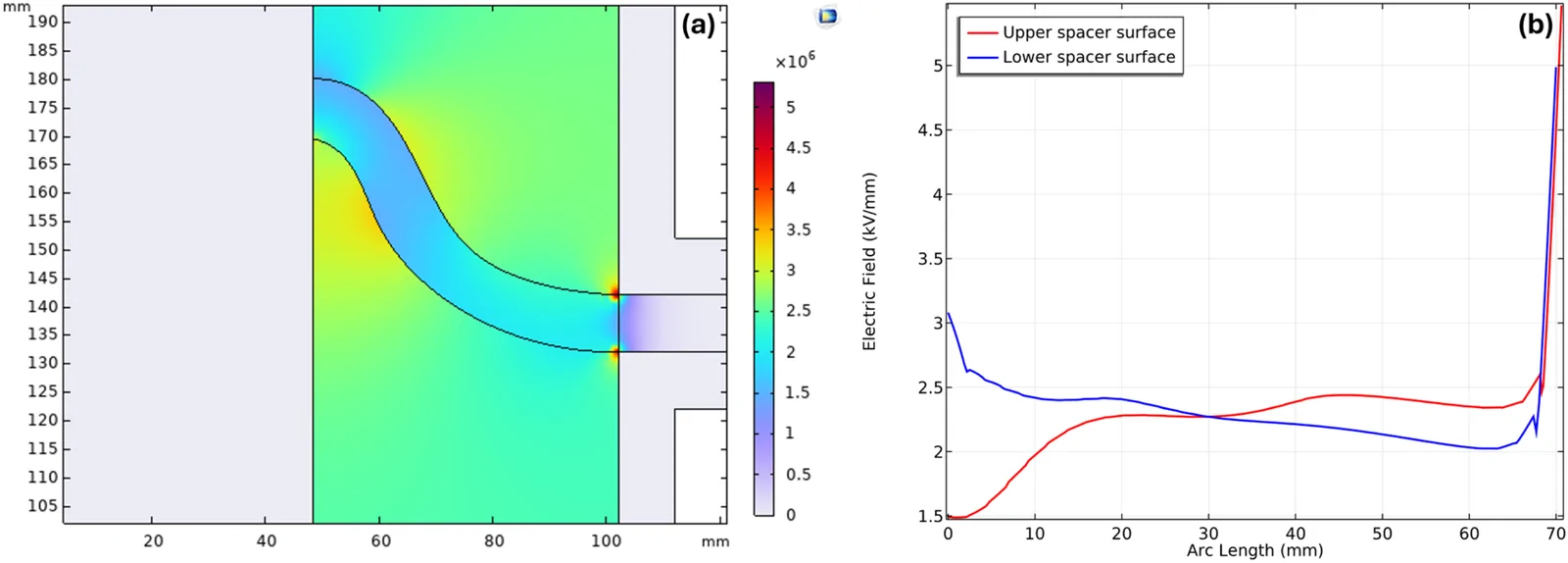
(a) Field map along the spacer surface and its enclosure, (b) electric field distribution along the upper and lower surfaces of the spacer.

The simulated electric field distribution of the pure epoxy spacer under DC excitation is presented in Fig. 20. The field remains relatively uniform along most of the spacer arc, with values in the range of 2.0–2.5 kV mm^−1^, before rising sharply near the triple-junction regions on both the conductor and enclosure sides. Peak values of approximately 5.8 kV mm^−1^ on the upper surface and 4.9 kV mm^−1^ on the lower surface confirm the expected field crowding at these geometrically critical locations, which represent the principal sites for charge localization and flashover initiation under long-term DC stress. This baseline distribution establishes the reference condition against which the nanocomposite improvements are evaluated.

**Fig. 21:**
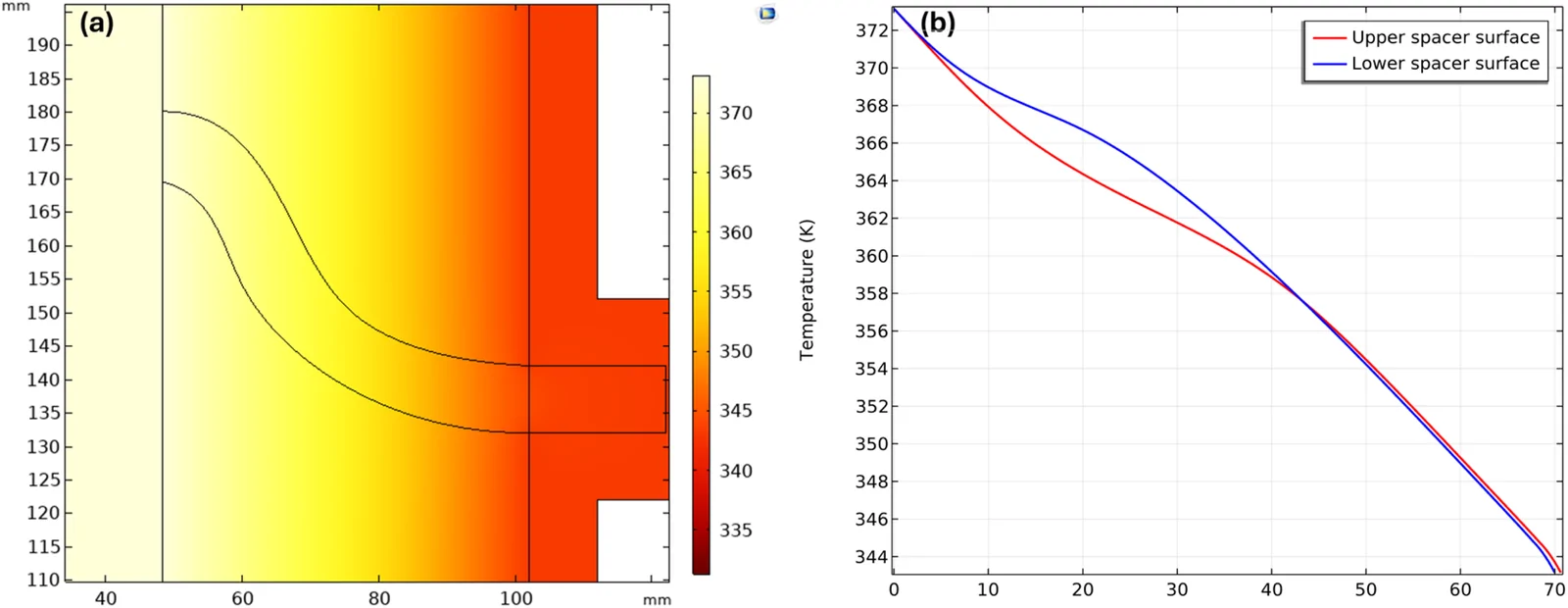
(a) Heat map along the spacer surface and its enclosure, (b) temperature distribution along the upper and lower surfaces of the spacer.

The thermal boundary conditions 105 °C at the conductor interface and 70 °C at the grounded enclosure produce a smooth and continuous temperature gradient across the spacer body, as shown in Fig. 21. This gradient activates the thermally sensitive conductivity model throughout the spacer volume and introduces spatial variations in charge transport rate that drive the time-dependent surface charge accumulation.

**Fig. 22:**
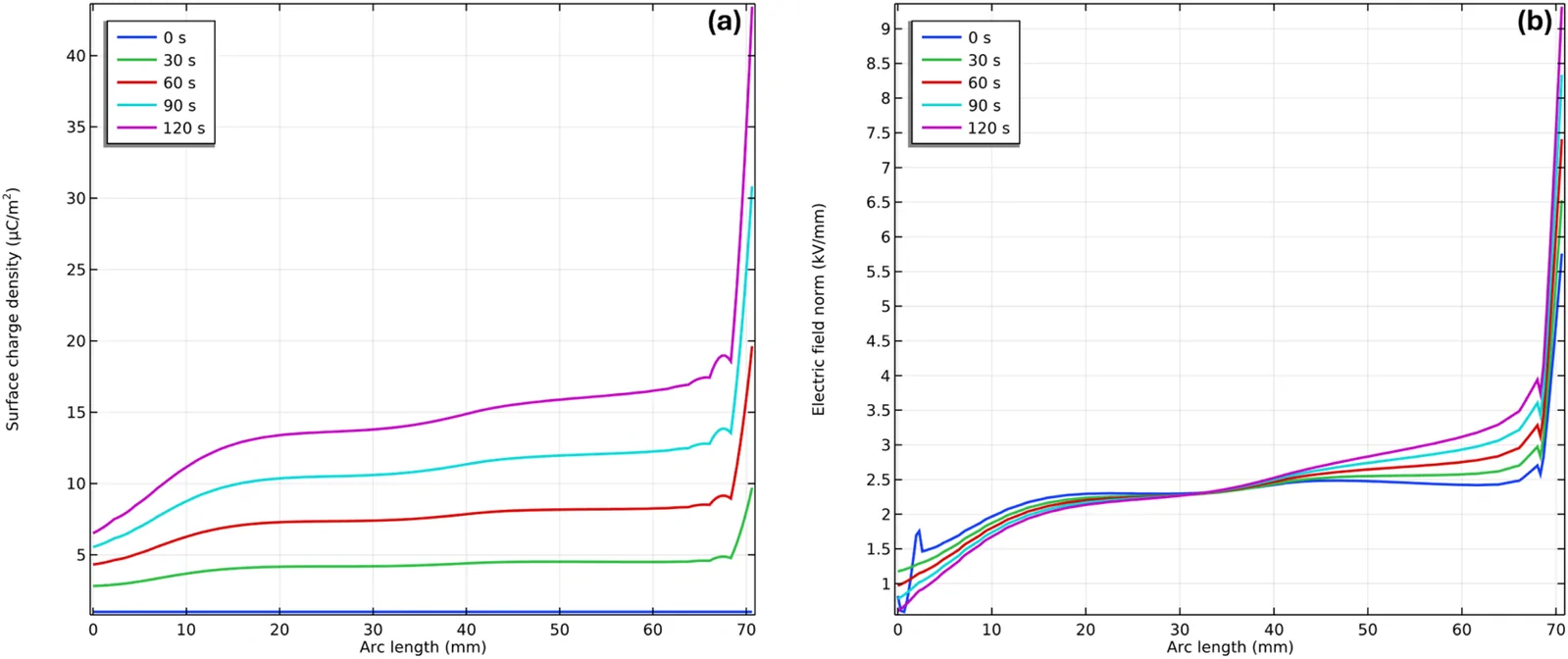
(a) Surface charge density accumulation, and (b) electric field intensity over the spacer arc length for pure epoxy.

The temporal evolution of surface charge density and the corresponding electric field along the spacer surface are presented in Fig. 22, and 23 for pure epoxy and both alumina nanocomposites respectively and summarized in Table 7.

**Fig. 23:**
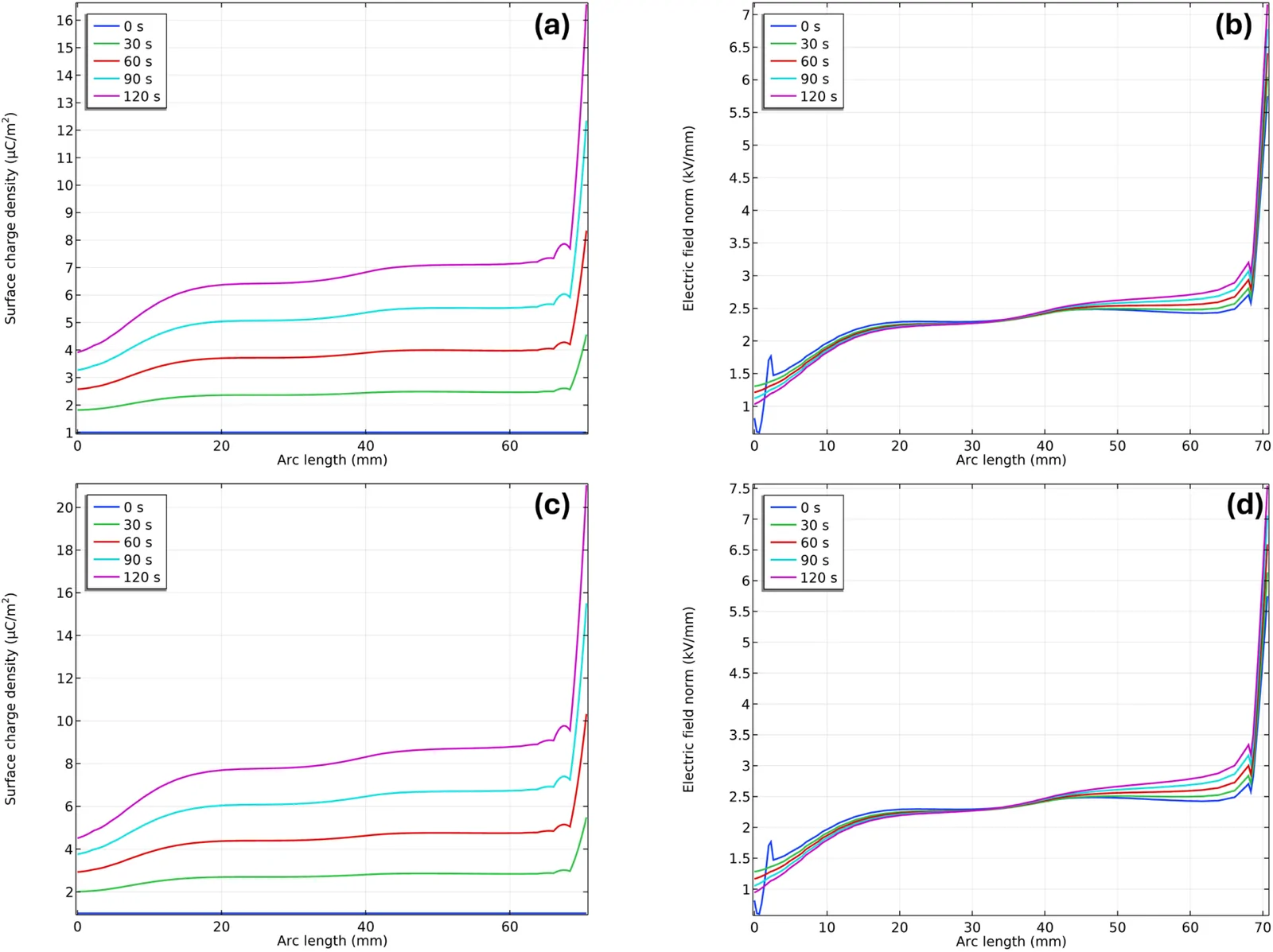
(a) Surface charge accumulation for γ-rich alumina nanocomposite, (b) electric field distribution for γ-rich alumina nanocomposite, (c) surface charge accumulation for α-Al_2_O_3_ nanocomposite, (d) electric field distribution for α-Al_2_O_3_ nanocomposite.

**Table 7:** Maximum surface charge density and maximum electric field intensity at 120 s for pure epoxy and the optimum nanocomposite systems used in the GIS spacer simulation

Material	Charge density at 120 s		*E*_max_ at 120 s	
	Value (µC m^−2^)	Reduction (%)	Value (kV mm^−1^)	Reduction (%)
Pure epoxy	43.40	—	9.30	—
γ-Rich alumina	16.58	61.8	7.15	23.1
α-Al_2_O_3_	21.05	51.5	7.55	18.8

For pure epoxy, surface charge accumulated progressively with charging time, reaching a maximum density of 43.40 µC m^−2^ after 120 seconds, with strong localization near the triple-junction region. As a direct consequence, the peak electric field increased from the initial 5.8 kV mm^−1^ to 9.30 kV mm^−1^ after 120 seconds which is an amplification of more than 60%. This result demonstrates the severe field distortion produced by accumulated surface charge in the unmodified epoxy spacer.

Both alumina nanocomposites produced substantially lower charge accumulation levels. γ-Rich alumina achieved the lowest surface charge density among all compositions investigated in this study, reaching only 16.58 µC m^−2^ at 120 seconds, corresponding to a 61.8% reduction relative to pure epoxy. The associated peak electric field was suppressed to 7.15 kV mm^−1^ corresponding to a 23.1% reduction. α-Al_2_O_3_ produced intermediate but still significant improvements, with a maximum charge density of 21.05 µC m^−2^ (51.5% reduction) and a peak electric field of 7.55 kV mm^−1^ (18.8% reduction).

The simulation performance ranking, γ-rich alumina > α-Al_2_O_3_ > pure epoxy, is qualitatively consistent with the experimentally measured surface-resistivity ranking. However, the measured surface resistivity was not converted into the bulk–conductivity parameter used in the COMSOL domain. The COMSOL model employed an effective bulk conductivity described by eqn (11), whereas surface resistivity was used only as an independent experimental indicator of charge transport along the gas–solid interface. Therefore, the agreement between the experimental and simulated rankings should be interpreted as qualitative consistency between the two datasets rather than as a direct parameter translation.

## 4. Study limitations

Several limitations of the present study should be acknowledged. First, all experimental characterization was conducted at near-ambient temperature; the influence of elevated operating temperatures on trap energy distributions, surface resistivity, and DC flashover performance was not directly measured, and the temperature dependence of the investigated nanocomposites was inferred from the electrothermal simulation model rather than from systematic experimental data at GIS-relevant thermal conditions. Second, the DC surface flashover tests were performed on pre-discharged specimens, which minimizes the contribution of accumulated charge history and may not fully represent the pre-charged surface conditions that develop during sustained HVDC service. Finally, system-level performance was assessed through numerical simulation of a reduced-scale 2D axisymmetric proof of concept model; full-scale experimental validation through prototype fabrication and high-voltage type testing was not conducted.

## 5. Conclusions

This study presented a systematic multi-scale comparison of γ-rich alumina and α-Al_2_O_3_ nanofillers derived from recycled aluminum beverage cans, incorporated into epoxy at 1–7 wt%, for HVDC GIS spacer surface insulation. The principal conclusions are as follows:

(1) Among the investigated filler concentrations, 5 wt% produced the highest surface resistivity for both alumina phases and was therefore selected for subsequent detailed characterization. This selection was specific to the surface-resistivity response and should not be interpreted as a universal optimum across all electrical performance variables. The decrease observed at 7 wt% was consistent with the increased particle agglomeration identified by SEM, particularly for the α-Al_2_O_3_ composite.

(2) γ-Rich alumina achieved the highest surface resistivity improvement of 68.1% (3.58 × 10^12^ Ω per sq) and the highest numerical mean DC flashover voltage of 13.49 kV (+12.8%), however, its difference from α-Al_2_O_3_ was not statistically significant (*p*-value = 0.23). Therefore, the two alumina nanocomposites exhibited statistically comparable flashover performance under the present experimental condition. These results are attributed to the larger specific surface area of the γ phase (≈196.5 m^2^ g^−1^), which provides a more extensive interfacial footprint and a higher deep trap density that suppresses surface carrier transport.

(3) α-Al_2_O_3_ exhibited the highest early-stage potential dissipation of 44.0% within 15 minutes, attributable to its markedly elevated shallow trap density (3.571 × 10^15^ eV^−1^ m^−3^), more than double that of γ-rich alumina. Its higher Weibull shape parameter indicates more consistent and predictable flashover behavior.

(4) Integrated mechanistic interpretation identified surface resistivity as the primary mechanism governing flashover performance under pre-discharged laboratory testing conditions, with the numerical flashover ranking exactly reproducing the surface resistivity hierarchy. Trap-mediated charge dissipation constitutes a supporting mechanism whose relative importance is expected to increase under sustained HVDC service conditions.

(5) System-level COMSOL simulation confirmed that γ-rich alumina reduced accumulated surface charge by 61.8% and suppressed the peak triple-junction electric field by 23.1% after 120 s of DC charging.

## Author contributions

Mahmoud Ezzat: methodology, formal analysis, investigation, data curation, writing – original draft, visualization. M. Ramadan: conceptualization, methodology, formal analysis, writing – original draft & review, supervision. Musa A. Said: supervision, resources, validation. Hany M. Abd El-Lateef: supervision, resources, validation. Mousa A. Abd-Allah: conceptualization, supervision, resources, validation, writing – review & editing. S. M. A. El-Gamal: conceptualization, supervision, resources, validation, writing – review & editing. Abdelrahman Said: conceptualization, supervision, validation, writing – review & editing.

## Conflicts of interest

The authors declare no competing interests.

## Data Availability

The data supporting the findings of this study are available from the corresponding author upon reasonable request.

## References

[cit1] Tang B., Xu Y., Zhuo R., Xiong J., Tang J. (2026). Coatings.

[cit2] Shengtao L., Mingru L. (2024). iEnergy.

[cit3] Adnan M. M. , Tveten E. G., Glaum J., Ese M. H. G., Hvidsten S., Glomm W. and Einarsrud M. A., Epoxy-Based Nanocomposites for High-Voltage Insulation: A Review, Blackwell Publishing Ltd, 2019

[cit4] Li Z., Liu J., Ohki Y., Chen G., Gao H., Li S. (2023). High Voltage.

[cit5] Wang T., Li X., Zhang B., Li D., Liu J., Zhang G. (2022). Sci. China Mater..

[cit6] Ramadan M., Ezzat M., Abd-Allah M. A., El-Gamal S. M. A., Said A. (2026). Sci. Rep..

[cit7] Ezzat M., Said A., Abd-Allah M. A., El-Gamal S. M. A., Ramadan M. (2026). J. Mater. Sci.: Mater. Electron..

[cit8] Wang Z., Liu C., Cao X., Cheng B., Wang J., Sun G. (2026). Adv. Funct. Mater..

[cit9] Jia J., Lin X., Geng Z., Xu J. (2025). Appl. Sci..

[cit10] Liang F., Wang F., Chen S., Sun Q., Zhong L. (2023). High Voltage.

[cit11] Chillu N., Jayaganthan R., Rao B. N., Danikas M., Tanaka T., Sarathi R. (2020). IET Sci., Meas. Technol..

[cit12] Aslam F., Li Z., Qu G., Feng Y., Li S., Li S., Mao H. (2021). Materials.

[cit13] Wu J., Zhang B., Li T., Du Y., Cao W., Yang H. (2022). Polymers.

[cit14] Ul Haq I., Wang F. (2023). IET Nanodielectr..

[cit15] Lian H., Yan J., Xie J., Song Y., Zhou H., Kang Y., Fangcheng L., Xie Q. (2020). Polym. Compos..

[cit16] Liu K., Zhang F., Liu Z., Song C., Zhang L., Ming W., Yang L., Wang Y., Huang B., Li J. (2024). Small Methods.

[cit17] Li Z., Liu J., Ohki Y., Chen G., Li S. (2025). High Voltage.

[cit18] Sukesh Babu M., Sarathi R., Jayantilal Vasa N., Imai T. (2020). High Voltage.

[cit19] Dąda A., Błaut P., Kuniewski M., Zydroń P. (2023). Energies.

[cit20] Xie Z., Pang X., Xu T., Liu P., Wei D., Wang J., Wu Z., Li H., Peng Z. (2023). J. Phys. D:Appl. Phys..

[cit21] Siddique A., Khan M. M., Aslam W., Shahid M. U., Alatawi K. S. S., Altimania M. R., Zaitsev I., Munir H. M. (2025). Energy Sci. Eng..

[cit22] Farrokhnemoun D., Sajjadivand S., Parapari S. S., Sturm S., Ow-Yang C. W., Gulgun M. A. (2024). J. Am. Ceram. Soc..

[cit23] Bu Z., Xue Y., Zhao X., Liu G., An Y., Zhou H., Chen J. (2024). ACS Appl. Mater. Interfaces.

[cit24] Sangor F. I. M. S., Al-Ghouti M. A. (2023). Case Stud. Chem. Environ. Eng..

[cit25] Sharma B., Sahi A., Dhau J., Kaushik A., Kumar R., Chaudhary G. R. (2024). Mater. Adv..

[cit26] Rahman M. L., Islam M. S., Ahmed M. F., Biswas B., Sharmin N., Neger A. J. M. T. (2023). Arabian J. Chem..

[cit27] Said A., Ezzat M., Abd-Allah M. A., El-Gamal S. M. A., Ramadan M. (2026). Electr. Power Syst. Res..

[cit28] Asaad J., Gomaa E., Bishay I. K. (2008). Mater. Sci. Eng. A.

[cit29] Aminifazl A., Karunarathne D. J., Golden T. D. (2024). Molecules.

[cit30] ASTM International , ASTM D257-14(2021)e1: Standard Test Methods for DC Resistance or Conductance of Insulating Materials, 2021, 10.1520/D0257-14R21E01

[cit31] Potocnik V. , Sarhan A. and Khan A., Measurement of Temperature Dependence of Electrical Insulation Resistance of Epoxy Grout and Two Industrial Laminates for Busbar Insulation, 2018

[cit32] Wang T., Liu C., Li D., Hou Y., Zhang G., Zhang B. (2020). IEEE Trans. Dielectr. Electr. Insul..

[cit33] Zebouchi N., Li H., Haddad M. A. (2020). Energies.

[cit34] Li H., Zebouchi N., Haddad M., Reid A., Ekkel E. (2022). Energies.

[cit35] Cheng L., Ma H., Liu X., Tang Q., Yang K., Zhang Y., Zhang H., Wang H., Xiong S. (2025). Chem. Eng. J..

[cit36] Wang X., Pakdel A., Zhang J., Weng Q., Zhai T., Zhi C., Golberg D., Bando Y. (2012). Nanoscale Res. Lett..

[cit37] Tsen A. W., Brown L., Levendorf M. P., Ghahari F., Huang P. Y., Havener R. W., Ruiz-Vargas C. S., Muller D. A., Kim P., Park J. (2012). Science.

[cit38] Manjumol C. C., Limna Mol V P., Deepa B., Sabukuttan D., Nevin K. G. (2025). Bionanoscience.

[cit39] Sypabekova M. , Hagemann A., Rho D. and Kim S., Review: 3-Aminopropyltriethoxysilane (APTES) Deposition Methods on Oxide Surfaces in Solution and Vapor Phases for Biosensing Applications, 2023 10.3390/bios13010036 PMC9856095 36671871

[cit40] Moyo D. S., Kleinhans G., Wei X., Doucet F. J., van der Merwe E. M. (2025). Minerals.

[cit41] Ezzat M., Ramadan M., Said M. A., Abd El-Lateef H. M., Abd-Allah M. A., El-Gamal S. M. A., Said A. (2026). J. Mater. Res. Technol..

[cit42] Arunarajeswari P., Mathavan T., Jeyaseelan S. C., Divya A., Benial A. M. F. (2022). Chem. Data Collect..

[cit43] Matějová L., Šolcová O., Schneider P. (2008). Microporous Mesoporous Mater..

[cit44] Da Ros S., Barbosa-Coutinho E., Schwaab M., Calsavara V., Fernandes-Machado N. R. C. (2013). Mater. Charact..

[cit45] Huan X., Hu B., Ji Z., Gao C., Zhou F., Yu J., Du B., Zhao Y. (2025). Compos. Commun..

[cit46] Simunin M. M., Voronin A. S., Fadeev Y. V., Mikhlin Y. L., Lizunov D. A., Samoilo A. S., Chirkov D. Y., Voronina S. Y., Khartov S. V. (2021). Polymers.

[cit47] Janaki G. B., Xavier J. R. (2021). Surf. Coat. Technol..

[cit48] Liu Q., Du B., Mai Y., Zhao Y. (2022). J. Electron. Mater..

[cit49] Pfeifer S., Bandaru P. R. (2014). Mater. Res. Lett..

[cit50] Khare H. S., Burris D. L. (2010). Polymer.

[cit51] Wang W., Li C., Huang W., Hu L., Chen G., Feng Y., Li S. (2026). Polymer.

[cit52] Khan M. Z., Aslam F., Alsaif F. (2025). Sci. Rep..

[cit53] Cheng X., He G., Sun Z., Wang Y., Geng S., Lian H. (2023). J. Phys. D:Appl. Phys..

[cit54] Du B., Li J., Li Z., Li A. (2018). IET Sci., Meas. Technol..

[cit55] Dai C., Liu N., Zhang Z., Ding M., Paramane A., Teng C. (2026). Polym. Compos..

[cit56] Wang X., Wang Z., Chen J., Shi X., Li X. (2022). Energies.

